# Transcriptomic Signatures of Ageing Vary in Solitary and Social Forms of an Orchid Bee

**DOI:** 10.1093/gbe/evab075

**Published:** 2021-04-29

**Authors:** Alice Séguret, Eckart Stolle, Fernando A Fleites-Ayil, José Javier G Quezada-Euán, Klaus Hartfelder, Karen Meusemann, Mark C Harrison, Antonella Soro, Robert J Paxton

**Affiliations:** 1 Institute of Biology, Martin-Luther-University Halle-Wittenberg, Halle, Germany; 2 Institute for Evolution and Biodiversity, Westfälische-Wilhelms University, Münster, Germany; 3 Leibniz Institute of Animal Biodiversity, Zoological Research Museum Alexander Koenig, Center of Molecular Biodiversity Research, Bonn, Germany; 4 Department of Apiculture, Campus of Biological Sciences and Animal Husbandry, Autonomous University of Yucatán, Mérida, Mexico; 5 Ribeirão Preto School of Medicine, University of São Paulo, Ribeirão Preto, Brazil; 6 Evolutionary Biology and Ecology, Albert-Ludwigs-University Freiburg, Freiburg (i. Brsg.), Germany; 7 German Centre for Integrative Biodiversity Research (iDiv) Halle-Jena-Leipzig, Leipzig, Germany

**Keywords:** gene expression, juvenile hormone, ageing, Hymenoptera, *Euglossa*, facultative sociality

## Abstract

Eusocial insect queens are remarkable in their ability to maximize both fecundity and longevity, thus escaping the typical trade-off between these two traits. Several mechanisms have been proposed to underlie the remolding of the trade-off, such as reshaping of the juvenile hormone (JH) pathway, or caste-specific susceptibility to oxidative stress. However, it remains a challenge to disentangle the molecular mechanisms underlying the remolding of the trade-off in eusocial insects from caste-specific physiological attributes that have subsequently arisen. The socially polymorphic orchid bee *Euglossa viridissima* represents an excellent model to address the role of sociality per se in longevity as it allows direct comparisons of solitary and social individuals within a common genetic background. We investigated gene expression and JH levels in young and old bees from both solitary and social nests. We found 902 genes to be differentially expressed with age in solitary females, including genes involved in oxidative stress, versus only 100 genes in social dominant females, and 13 genes in subordinate females. A weighted gene coexpression network analysis further highlights pathways related to ageing in this species, including the target of rapamycin pathway. Eleven genes involved in translation, apoptosis, and DNA repair show concurrent age-related expression changes in solitary but not in social females, representing potential differences based on social status. JH titers did not vary with age or social status. Our results represent an important step in understanding the proximate mechanisms underlying the remodeling of the fecundity/longevity trade-off that accompanies the evolutionary transition from solitary life to eusociality.

**Significance**

The remarkably long lifespan of the queens of eusocial insects despite their high reproductive output contradicts the widespread trade-off between fecundity and longevity that governs solitary animal life histories, yet surprisingly little is known of the molecular mechanisms underpinning their longevity. Using a socially polymorphic bee in which some individuals of a population are social whereas others are solitary, we identified hundreds of genes and related gene networks, including target of rapamycin and antioxidation pathways, that are involved in the remolding of the fecundity/longevity trade-off. Besides identifying candidate ageing genes, our data suggest that even in incipient stages of sociality there is already a marked reprogramming of ageing; long live the queen.

## Introduction

There is longstanding empirical support for the costs of reproduction, and more specifically for the trade-off between fecundity and longevity (Maynard-Smith 1958). Typically, investment in the production and care of offspring has a negative effect on survival (Edward and Chapman 2011). Evidence for such a trade-off has been found in organisms ranging from zebra finches (Alonso-Alvarez et al. 2004) to Columbian ground squirrels (Festa-Bianchet and King 1991), with much support found in model systems such as *Drosophila melanogaster* (Flatt 2011).

Previous studies have helped identify some of the proximate mechanisms underlying this widespread negative correlation between reproduction and lifespan. Endocrine pathways play a role in this context, with juvenile hormone (JH) correlating positively with reproduction but negatively with lifespan in *Drosophila* (Flatt et al. 2005), in line with the antagonistic pleiotropy theory of ageing (Medawar 1952; Williams 1957). Additionally, oxidative stress, a molecular marker of senescence according to the free radical theory of ageing (Harman 1956), has been proposed as a proximate mechanism mediating the trade-off between reproduction and lifespan, with reproduction leading to a decrease in antioxidant defenses in zebra-finches (Alonso-Alvarez et al. 2004), and increased egg production inducing increased susceptibility to oxidative stress in *D. melanogaster* (Wang et al. 2001). Another widespread life history mediator is nutrient-sensing, more specifically the insulin/insulin-like signaling pathway and the target of rapamycin (TOR) pathway (Kapahi et al. 2010). These pathways mediate the link between reproductive investment and lifespan in many organisms, including *D. melanogaster* (Flatt et al. 2008), *Caenorhabditis elegans* (Kenyon 2010), and humans (Blagosklonny 2010).

Although evidence for a trade-off between reproduction and longevity is abundant, controversy remains. Germ-line ablation did not cause differences in lifespan in *D. melanogaster* males, and even slightly decreased lifespan in females (Barnes et al. 2006). In addition, the insulin/IGF1 pathway, previously thought central in mediating the fecundity/longevity trade-off across animals, has been shown to control longevity and fecundity independently of each other in *C. elegans* (Dillin et al. 2002).

Adding to this controversy, a major challenge to the existence of a universal fecundity/longevity trade-off is the remarkable case of eusocial insects, where queens are often the only reproductively active individuals in their colony, yet they live up to 30 times longer than their worker nestmates (Carey 2001). Reproductive individuals in species exhibiting complex eusocial behavior such as the holometabolous honeybees, stingless bees, several wasps and all ants, as well as the hemimetabolous eusocial termites, all exhibit a clearly positive and not a negative correlation between reproduction and lifespan (Page and Peng 2001). This remolding of the expected longevity/fecundity trade-off in eusocial insects has also been supported experimentally. In the ant *Cardiocondyla obscurior*, mating appears to incur no cost in terms of longevity, as mated queens live longer than virgin queens (Schrempf et al. 2005), and enforced changes in egg-laying rate did not affect the longevity of queens (Schrempf et al. 2017). Moreover, in honeybees, workers which develop under queenless conditions have higher reproductive potential and also live longer than workers developing in queenright colonies, thus seemingly circumventing the trade-off between fecundity and longevity (Kuszewska et al. 2017).

Several molecular mechanisms have been suggested to underlie the apparent remolding of the fecundity/longevity trade-off in insects exhibiting complex eusocial behavior compared with solitary insects and other solitary organisms. For instance, caste-specific differences in somatic maintenance have been observed in ants, with *Lasius niger* queens exhibiting higher expression of somatic repair genes than workers (Lucas et al. 2016). Evidence suggests that a rewiring of central endocrine pathways may underlie the remolding of the trade-off. Specifically, titers of JH and vitellogenin (the primary yolk protein precursor) are both positively correlated with reproduction at the expense of longevity in solitary insects, but the JH/vitellogenin network connectivity varies considerably in social insects (Rodrigues and Flatt 2016). In *Apis mellifera*, for instance, JH and vitellogenin are not connected in queens, as JH titers are low throughout their adult life, whereas vitellogenin levels increase rapidly as they start to lay eggs and remain at high levels all throughout their reproductive life (Hartfelder and Engels 1998). In workers, JH and vitellogenin are even negatively connected, and the increase in JH levels once workers become foragers actually sets a limit on their adult lifespan by promoting immunosenescence (Amdam et al. 2005). Hence, in *A. mellifera* queens and workers, vitellogenin is seemingly positively related to somatic maintenance and thus longevity, with high vitellogenin levels making them more resistant to oxidative stress (Seehuus et al. 2006; Corona et al. 2007). By circumventing the positive correlation between JH and vitellogenin of solitary insects, and given the positive impacts of vitellogenin on longevity in the honeybee, queens of this species are thus able to maintain high reproductive function without sacrificing longevity. In contrast, recent findings suggest that in the primitively eusocial and phylogenetically close-related *Bombus terrestris*, JH has retained its role as a major gonadotropin, which comes at a cost to brain function (Shpigler et al. 2020). Although a multitude of mechanisms that promote queen longevity have been proposed in eusocial insects, we still lack a clear understanding of when and how this apparent remolding of the fecundity/longevity trade-off evolved (Toth et al. 2016).

Within Hymenoptera, eusociality along with a remolding of a fecundity/longevity trade-off has been suggested to have evolved independently at least eight times (Peters et al. 2017), yet studies have rarely investigated the fecundity/longevity trade-off in insect species across the sociality gradient, in particular primitively social and socially polymorphic species. In one exception, the reversal of the trade-off was shown to be evident in primitively social wasps (Toth et al. 2016), shedding light on the mechanisms at play during early stages of social evolution within wasps, such as caste-specific modulation of reproduction by JH (Kapheim 2017). We lack data on other species at the earliest stages of sociality. Socially polymorphic species such as the hover wasp *Parischnogaster alternata* (Bolton et al. 2006), the sweat bee *Megalopta genalis* (Kapheim et al. 2013), or the orchid bee *Euglossa viridissima* represent excellent study organisms for such investigations; the behavioral plasticity known for these species allows a direct comparison of solitary and social individuals within the same species, or even within the same population. Such comparisons allow us to identify molecular mechanisms underlying the remolding of the trade-off in early stages of social evolution, thus distinguishing them from mechanisms that maintain or reinforce this remolding in complex insect societies (Séguret et al. 2016; Shell and Rehan 2018).

**Fig. 1:**
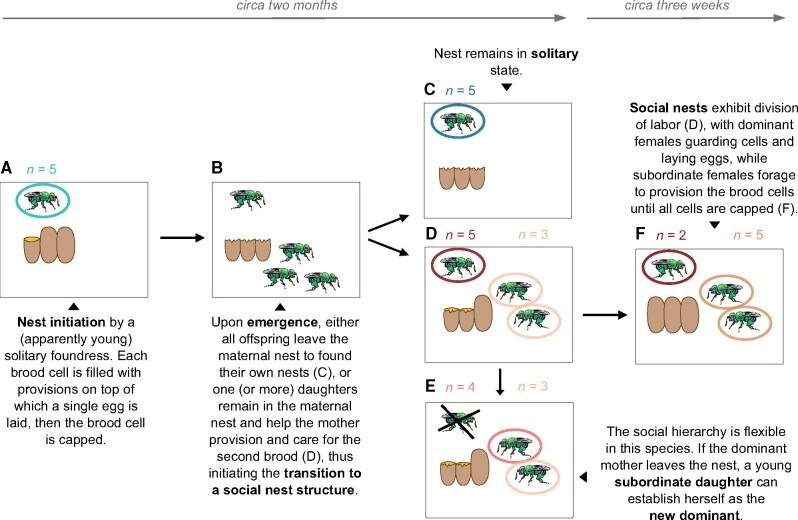
Lifecycle of *Euglossa viridissima* and sampling design. Colored circles represent the different age and female types (solitary, social dominant, or subordinate) sampled for this study. Young solitary females (light blue) were sampled from nests as shown in (*A*). Upon emergence of the first brood (*B*), old solitary females (dark blue) were sampled from nests as shown in (*C*); old dominant (dark red) and young subordinate females (yellow) from social nests as shown in (*D*); old dominant (dark red) and old subordinate females (brown) from social nests as shown in (*F*); and young dominant (light red) and young subordinate females (yellow) from nests as shown in (*E*), where the old dominant had left the nest and one of the previously subordinate daughters established herself as dominant. Sample sizes for each nest stage are shown above the nests, color-coded same as the circles according to the social status and age of the female.

The Neotropical orchid bee *E. viridissima* Friese, 1899, is facultatively eusocial, with solitary and social nests co-occurring within the same population (Cocom-Pech et al. 2008; May-Itzá et al. 2014). All nests established by a “foundress” female are initially solitary until the first brood emerges approximately 2 months after founding (fig. 1). Nests are commonly reused for a second brood and can be reactivated as a single-female nest (solitary) or a multifemale nest in which one or more daughters from the first brood remain in the natal nest and share in care of future broods (social nests). In such multifemale nests, a dominance hierarchy is established through aggressive behavior, with reproductive skew in favor of the mother (Cocom-Pech et al. 2008).

In this study, we aimed to determine how social organization and reproductive status (caste) influence lifespan in *E. viridissima* females. Specifically, we investigated changes in gene expression and JH titer with age in reproductive individuals from solitary nests and compared these with observed changes with age in dominant and subordinate females from social nests. Though there are discrepancies in JH patterns in relation to age and reproductive status across the bees and wasps (Huang and Robinson 1996; Kelstrup et al. 2015; Cardoso-Júnior et al. 2017), we were interested in discovering whether *E. viridissima* patterns were similar to those typical for solitary insects such as *D. melanogaster* (Flatt et al. 2005) or rather were independent of reproductive function as in the solitary wasp *Synagris cornuta* (Kelstrup et al. 2015) and in highly eusocial relatives such as *A. mellifera* (Huang and Robinson 1996). Regarding the gene expression profiles of *E. viridissima* females relative to age and sociality, our aim was to test whether pathways outlined in the literature as being involved in the reshaping of the fecundity/longevity trade-off in eusocial insects (e.g., oxidative stress, somatic repair, and nutrient sensing) were associated with age-related differentially expressed genes (DEGs) in our study more than expected by chance. To address this, we undertook a differential expression analysis to identify genes with age-dependent expression in solitary and social females, followed by a functional enrichment analysis of these genes.

## Materials and Methods

### Experimental Setup and Sample Collection

All *E. viridissima* samples were collected at the Department of Apiculture of the Campus of Biological Sciences and Animal Husbandry, Autonomous University of Yucatán in Xmatkuil, Mexico (89.37°W, 20.52°N, sample collection permit no. 41593). Wooden boxes (7 × 3 × 3 cm) with an inner coating of beeswax and stingless bee cerumen were placed around the campus, with a glass cover between the box and wooden lid to facilitate observations. Females from the wild observed constructing cells in or bringing back provisions to a nest box were individually marked on the thorax with a diamond-tipped pen. Nests were checked three times weekly from February 2016 until June 2018 to record the presence of marked females. In multifemale nests, the hierarchy of the females (dominant, subordinate) was determined by observing nests until individuals could be classified based on behavior (supplementary table S1, Supplementary Material online). For multifemale nests with only two females, observations continued until a female was observed returning to the nest with pollen, which characterized this female as the subordinate. In multifemale nests with three or more females, the individual spending most time in the nest, on the brood cells, and exhibiting the largest number of aggressive behaviors toward other females was characterized as dominant (Boff et al. 2015).

**Fig. 2:**
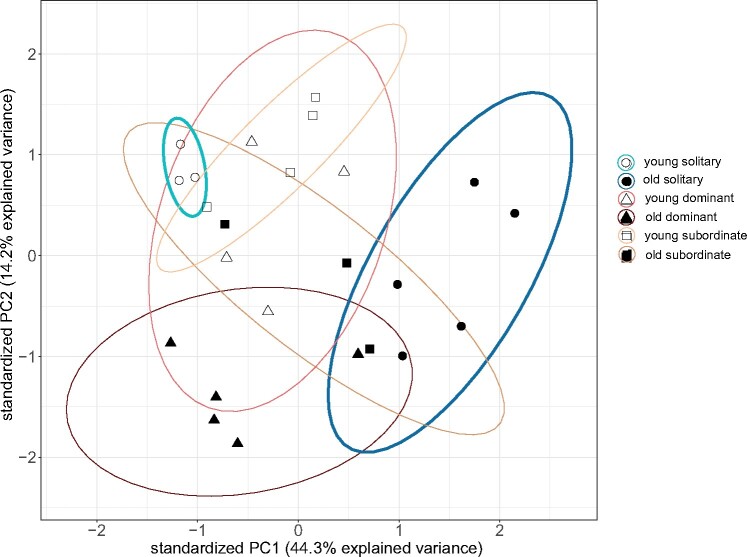
Principal component analysis (PCA) of variance-stabilized RNA read counts of young and old *Euglossa viridissima* females from solitary and social nests. Each point represents the expression profile of one individual across the 1,015 genes which were differentially expressed between young and old individuals, cumulative across all social types (solitary: 902 DEGs, dominant: 100 DEGs, subordinate: 13 DEGs, with five DEGs shared between solitary and subordinate, seven DEGs shared between solitary and dominant, and one DEG shared between dominant and subordinate, see figs. 3 and 4). Axis labels indicate the amount of variance in gene expression explained by the first two principal components (PC1 and PC2). Ellipses represent 95% confidence levels, and colors further illustrate the social type and age of each individual as in figure 1. For solitary females, gene expression profiles did not overlap between young and old individuals (thicker ellipses shown in blue) unlike for dominant or subordinate females.

For females from solitary nests and dominant females from social nests, both young (<1.5 months since marking) and old (>1.5 months since marking) individuals were collected. The establishment of a threshold of 1.5 months was based on personal observations in the field and on general life history: we estimated the lifespan of solitary and dominant *E. viridissima* females to be of 2–4 months based on anecdotal observations of *E. viridissima* during previous studies of this species at the same campus sites and on what is known for the socially polymorphic *Euglossa melanotricha* (Andrade-Silva and Nascimento 2015). Since “worker” (subordinate female) lifespan was unknown for this species at the time of sampling, but is generally much shorter than queen lifespan in other eusocial hymenopteran species (Carey 2001; Séguret et al. 2016), we used 3 weeks as a threshold for distinguishing “young” versus “old” workers based on previous personal observations of this species at the same sites, and supported post hoc by comparison of transcriptomes (see Results and fig. 2). For illustration of the *E. viridissima* life cycle and details of our sampling design, see figure 1. Collection continued until at least five individuals were collected for each combination of age and social status, except for young dominant females which were rarely found in the field.

Upon collection of each individual, a hemolymph sample (1.5–4 μl) was collected from the abdomen using microcapillaries for JH measurements. Each hemolymph sample was transferred to Teflon capped GC-vials containing 500 μl acetonitrile that was then stored at −80 °C. Whole bees were then kept overnight at −80 °C and, once frozen, the abdomen was removed and transferred to RNAlater (Sigma–Aldrich) for storage at −80 °C. Altogether, 34 individuals were used for molecular analyses (supplementary table S2, Supplementary Material online).

### RNA Extraction and Sequencing

Total RNA was extracted from each individual (whole abdomen) using an RNeasy Plus Mini kit, including DNase I digestion, according to manufacturer’s instructions, using 350 μl starting material (Qiagen, Hilden, Germany). RNA concentration, purity, and integrity were measured using an Agilent 4200 TapeStation and six RNA ScreenTapes. Samples which passed our quality criteria (260/280 = 2.1 ± 0.1, RINe > 9, total RNA mass > 1 µg), including five young solitary females, five old solitary females, four young dominant females, seven old dominant females, six young subordinate females and five old subordinate females, as well as two dominant females of ambiguous age (later excluded from downstream analyses), were used for RNA sequencing (supplementary table S2, Supplementary Material online). Library preparation and transcriptome sequencing were undertaken at the Beijing Genomics Institute (BGI, Shenzhen). For each sample, a cDNA library was prepared with the TruSeq RNA Library Preparation kit (Illumina) and SuperScript II Reverse Transcriptase (Thermo Fisher Scientific). Libraries were sequenced on an Illumina HiSeq4000 platform (11 samples per lane) to generate 100-bp paired-end reads, with approximately 4 GB of raw data per sample, that is, 29 million read pairs per sample on average (range 24–35 million).

Raw reads were quality-checked with FastQC v0.11.5 (Andrews 2010), then filtered and trimmed using Skewer v0.2.2 (Jiang et al. 2014) to remove low-quality bases and reads, adapter contamination, and reads shorter than 70 bp.

### Exclusion of Individuals from a Sister Species

Individuals belonging to the cryptic sister species *Euglossa dilemma* were identified through sequence comparison of the olfactory receptor gene *or41*, which has been described as a means of differentiating between *E. viridissima* and *E. dilemma* (Brand et al. 2020) (supplementary table S3, Supplementary Material online). Six *E. dilemma* individuals were identified and subsequently excluded from further analysis in order to avoid any bias, although overall patterns in the data did not change when including these individuals (supplementary fig. S4 and tables S7 and S8, Supplementary Material online).

### Differential Gene Expression Analysis

#### Transcriptome Assembly, Genome Annotation, Read Pseudoalignment, and Quantification

For full details on the transcriptome assembly and genome annotation, as well as software references, see supplementary file S9, Supplementary Material online. The transcriptomic analyses are based on the previously published draft genome sequence assembly for *E. dilemma* GCA_002201625.1 (Brand et al. 2017). Repeats were soft-masked (35.30% of the total genome assembly length) using bedtools v2.27.1 based on repeat annotations from Tandem Repeats Finder v4.09 and RepeatMasker. To improve the previously published annotation of this genome, which was based solely on gene predictions and homology to *A. mellifera* proteins (Brand et al. 2017), we used Funannotate v1.8.1 (Palmer and Stajich 2020) with the previous gene annotation (edil.1.0.annotations.gff) and novel experimental evidence derived from our RNAseq data generated in this study, including 1) RNAseq paired-end reads aligned to the sister species *E. dilemma* genome with HiSat2 v2.1.0, 2) a transcriptome assembly with binpacker v1.1 from one sample and 3) a genome-guided transcriptome assembly from four samples with Stringtie2 v1.3.3b, as well as data from published studies, namely 4) an RNAseq data-based transcriptome assembly of *E. dilemma* using Trinity v1.5.1. Further, protein sequences from 11 related bee species (*Bombus impatiens*: GCF_000188095.2; *B. terrestris*: GCF_000214255.1; *A. mellifera*: GCF_003254395.2; *Melipona quadrifasciata*: GCA_001276565.1; *Eufriesea mexicana*: GCF_001483705.1, *Friseomelitta varia*: GCA_011392965.1, *Megachile rotundata*: GCF_000220905.1, *Habropoda laboriosa*: GCF_001263275.1, *Dufourea novaeabgliae*: GCF_001272555.1, *Megalopta genalis*: GCF_011865705.1, *Nomia melanderi*: GCF_003710045.1; Kapheim et al. 2015, 2019, 2020; Wallberg et al. 2019) and Uniprot (sprot) were used for homology-based evidence.

In brief, PASA was used to train gene predictions which were then performed using Genemark-ES, PASA v2.4.1, Snap v2006-07-28, glimmerHmm v3.0.4, Augustus v.3.3.3, and CodingQuarry v2.0. Predictions as well as additional, previous annotations were then integrated in Evidence Modeler v.1.1.1. Too short, gap-spanning, or repeat-overlapping gene models were removed and tRNA genes were detected with tRNAscan-SE v2.0.6. Previous gene models were then updated with PASA and Transdecoder v5.5.0 based on RNAseq evidence and expression levels of models at each locus. Genes were functionally annotated using PFAM v33.1, the UniProt database v2018_11, EggNog (eggnog_4.5/hmmdb databases: Arthropoda, Insecta, Hymenoptera, Drosophila), MEROPS v12.0, CAZYmes in dbCAN v7.0, BUSCO Hymenoptera models v3.0.2, Hymenoptera odb9, SignalP v4.1, and InterProScan5 v81.0. The final annotation contained models for 17,550 protein-coding genes (from 17,528 loci) and 144 tRNAs and was estimated to be 86.5% complete (BUSCO4 v4.1.4).

This set of 17,694 gene models (hereafter referred to as *E. dilemma* annotation v3) was then used for subsequent transcriptome analyses. Trimmed and filtered RNAseq reads were pseudoaligned to these gene models and were simultaneously quantified using Kallisto v0.46.2 (Bray et al. 2016). GO terms for 8,530 transcripts were extracted from GO terms assigned by the Funannotate pipeline and GO terms fetched for *E. dilemma* orthologs (determined with Broccoli, Derelle et al. 2020) in *A. mellifera* and its orthologs in *D. melanogaster* through Biomart Ensembl using the R package biomaRt (Durinck et al. 2009) and modified scripts from Colgan et al. (2019) (supplementary file S12, Supplementary Material online).

#### Differential Expression Analyses

In order to analyze differential gene expression between young and old individuals within each group (see below), we used the tximport package v1.16.1 (Soneson et al. 2015) in R v4.0.2 (R Core Team 2019) to import transcript-level read quantification data, and convert these to gene-level quantification values (supplementary table S10, Supplementary Material online). We then ran DESeq2 v1.28.1 (Love et al. 2014) with default settings (adjusted *P* value calculated to correct for multiple testing following the BH method; Benjamini and Hochberg 1995) to perform pairwise comparisons of gene expression profiles between young and old individuals of a given social status (i.e., solitary, social dominant, or social subordinate), and between individuals of different social status for a given age. DEGs were identified as those with an adjusted *P* value below 0.05. After plotting gene expression profiles of all individuals, excluding *E. dilemma* individuals, in a principal component analysis plot (PCA of variance stabilized read counts, with genes as columns and samples as rows in the PCA matrix, supplementary fig. S5, Supplementary Material online), individual eug6 was identified as an outlier and was excluded from gene expression analyses. We also excluded individual eug25 from gene expression analyses to avoid any confounding effects of nest of origin, as this individual was sampled from the same nest as eug26, and both eug25 and eug26 were social dominant. For remaining samples (hereafter referred to as “core individuals”), expression profiles did not cluster according to nest of origin, allowing us to exclude nest as a confounding factor (supplementary fig. S6, Supplementary Material online). All PCA figures were produced using the R package ggbiplot v0.55 (Vu 2011), with ellipse probabilities (if ellipses shown) set to 95% confidence level (ellipse.prob = 0.95). The final data set comprised 24 *E. viridissima* transcriptomes, ranging from three to five samples per age × social type combination (supplementary table S2, Supplementary Material online).

To identify the genes for which expression levels were affected by the interaction between age and social status, we performed a likelihood ratio test to contrast the following models in DESeq2: Full model (age + social_status + age: social_status), Reduced model (age + social_status). This contrast identified genes with a significant change in expression associated with the interaction of the two factors, age and social status.

### Weighted Gene Coexpression Network Analysis

#### Network Construction and Correlation of Modules with Phenotypic Traits of Interest

A weighted gene coexpression network analysis was carried out using the R package WGCNA v1.69 (Langfelder and Horvath 2008). The aims of this analysis were to 1) identify sets of coexpressed genes (modules), 2) calculate module eigengenes (i.e., values representative of the gene expression profile in a module) and correlate these with phenotypes of interest (here, social status and age), and finally 3) identify “hub genes,” that is, genes that were most highly connected, within modules which were significantly associated with the phenotypic trait of interest (here, age). Additionally, the network analysis was performed to identify age-related hub genes overlapping with those differentially expressed between young and old individuals. For this analysis, genes with read counts lower than 10 in 90% of the samples were removed, and raw read counts were transformed using the varianceStabilizingTransformation function in DESeq2, as recommended by Langfelder and Horvath (2008). The input table consisted of a matrix of 25 individuals across all social types and ages and 17,527 gene expression values (supplementary table S11, Supplementary Material online). Modules of coexpressed genes were defined using average linkage hierarchical clustering with the topological overlap-based dissimilarity measure, with parameters set according to recommendations of Zhang and Horvath (2005) and Langfelder and Horvath (2008) (soft thresholding power set to 9 as the lowest value for which scale-free topology fit index reached 0.85; minimum module size set to 30 genes; modules of highly coexpressed genes merged using a cut-off value of 0.2).

#### Hub Genes Correlated with Age and Social Status

Module eigengenes were correlated with two phenotypic traits: social status (separated into solitary, dominant, and subordinate) and age (separated into old and young). Hub genes from each module associated with age, that is, genes which were most highly connected within those modules (intramodular connectivity >0.75) were extracted and concatenated to create a list of hub genes associated with age.

### Overlap of Age-Related Genes from Two Independent Analyses

Genes found to be common to the list of hub genes associated with age in the WGCNA and the list of genes found to be differentially expressed with age were considered to be of particular interest. The functions of these overlapping genes were further investigated through enrichment of their gene ontology (GO) annotations (see below).

### Functional Annotation of Age-Related Genes

Functional annotation was performed on the following gene lists of interest: 1) genes up- or downregulated with age in each social type (solitary, social dominant, social subordinate), 2) genes whose expression was affected by the interaction between age and caste (likelihood ratio test), 3) hub genes from modules correlating with age in the WGCNA, 4) genes found to be related to age in both the differential expression and the network analysis, and 5) genes commonly up- or downregulated with age in both solitary and subordinate females (highlighting the transcriptomic costs of tasks performed by both solitary and subordinate females, e.g., foraging). GO terms which showed significant enrichment in each gene list of interest (Fisher’s exact test with FDR correction, adjusted *P *<* *0.1) were obtained using the R package topGO v2.40.0, focusing on the level “biological process.” Word clouds of significantly enriched GO terms were generated with the R package tagcloud v0.6.

Additionally, seven known longevity-related genes from the model species *D. melanogaster* (*5-hydroxytryptamine (serotonin) receptor 2 A, trachealess, I’m not dead yet, Insulin-like 1 receptor, snazarus, Enhancer of zeste*, and *methuselah*) were specifically investigated for age-related expression patterns in our data set. For this, *D. melanogaster* protein sequences were downloaded from FlyBase version FB2020_05 (Thurmond et al. 2019) and their *A. mellifera* homologs were identified using BlastP (NCBI Nucleotide Database 2004, database accessed December 20, 2020). Using the previously generated ortholog assignment between *A. mellifera* and *E. dilemma* (see section on genome annotation), we then identified corresponding *E. dilemma* transcript IDs.

### Deposition of Genome Annotation Files and Raw Sequence Reads

All raw sequence reads for each sample, including *E. dilemma* individuals, have been deposited in the NCBI Sequence Read Archive under Bioproject ID PRJNA636137. The updated genome annotation for *E. dilemma* has been deposited in Dryad (doi: 10.5061/dryad.2547d7wnh, see Data Availability).

### Juvenile Hormone Quantification and Analysis

The same individuals used for the transcriptomic analyses were also used to assess JH levels in the hemolymph by a radioimmunoassay. The protocols for JH extraction from the hemolymph samples in acetonitrile and preparation of the samples in the radioimmunoassay followed the detailed description for JH quantification in the honeybee (Hartfelder et al. 2013) and wasps (Kelstrup et al. 2018), using a JH-specific antiserum, tritiated [10-^3^H(N)]-JH III (specific activity 19.4 Ci/nmol, Perkin Elmer Life Sciences, Waltham, MA), and synthetic JH-III (Fluka, Munich, Germany) as the nonradioactive competitor. For the hemolymph JH titer calculations, we used a nonlinear four-parameter regression. After exclusion of *E. dilemma* individuals, 21 *E. viridissima* individuals were included in the analysis (supplementary file S2, Supplementary Material online).

All statistical analyses for JH were performed in R (v3.6.1). To investigate the effect of age and social status on JH titers, we performed a linear mixed model on our log-transformed data using the package lme4 v1.1-21 (Bates et al. 2015). A stepwise Akaike Information Criterion method for model selection was applied, with the best-fitting model including age and social status as fixed factors, and nest ID and date of collection as random factors. An ANOVA was then used to test for significant effects. To assess our statistical power to detect any differences in JH given our low sample size, we performed a power analysis using the R package pwr v1.3-0 (Champely et al. 2017).

## Results

### RNA Sequencing

After trimming and filtering, sequenced libraries contained on average 29 million read pairs (range = 24–35 million read pairs; SD = 3 million read pairs), of which 72% (range = 62–76%; SD = 3%) could be mapped to the *E. dilemma* genome v3. A total of 15,046 genes had at least one read count, representing 86% of annotated genes.

### Gene Expression Changes with Age in Solitary but Not in Social Females

We compared gene expression levels based on read counts per gene between young and old individuals within each social type (solitary, social dominant, and social subordinate) and found a total of 1,015 DEGs with age across all comparisons. A PCA of normalized read counts across genes which were differentially expressed between young and old individuals, cumulative across all social types (fig. 2), showed a clear distinction between the gene expression profiles of young and old solitary females across both principle component 1 (PC1 explaining 44% of the variance) and principle component 2 (PC2 explaining 14% of the variance) (for a PCA across all genes, not just those which were differentially expressed, see supplementary fig. S13, Supplementary Material online). In dominant and subordinate females from social nests, however, gene expression profiles did not show a clear pattern with respect to age; data points from young and old individuals overlapped (fig. 2).

**Fig. 3:**
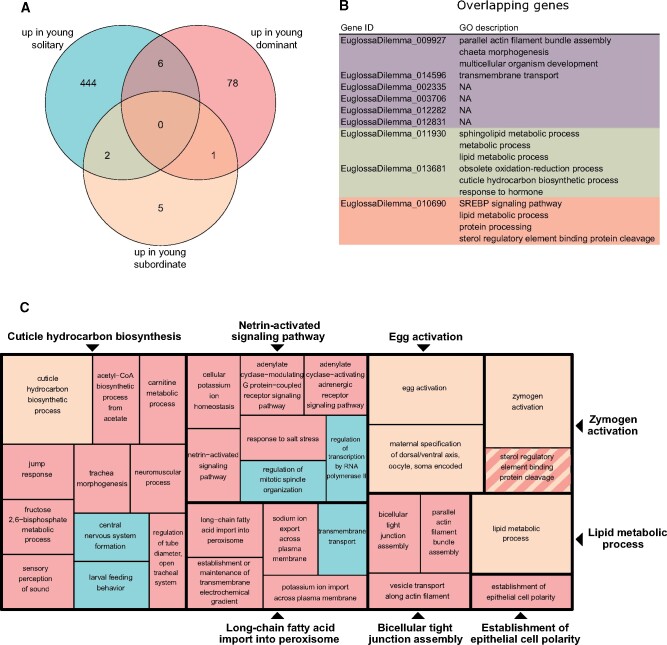
Venn diagram (*A*) and gene ontology (GO) annotations (*B* and *C*) for genes upregulated in young solitary, dominant, and subordinate *Euglossa viridissima* females (i.e., genes downregulated with age). Available GO annotations on the level Biological Process for genes differentially expressed in multiple comparisons are shown in (*B*) (NA, GO annotations not available), with colors corresponding to the different comparisons as in the Venn diagram (purple, up in young solitary/dominant; green, up in young solitary/subordinate; orange, up in young subordinate/dominant). A reduced visualization of GO terms for genes upregulated in the different comparisons is shown in (*C*), with GO terms clustered based on semantic similarity. Comparisons are color-coded as in (*A*). Each box represents a semantic cluster, and the size of each box is inversely proportional to the adjusted *P* value for the enrichment of GO terms within its cluster. Boxes outlined in bold represent “superclusters” of loosely related GO terms. Two singletons are represented in their own boxes, as they were not assigned to any supercluster.

**Fig. 4:**
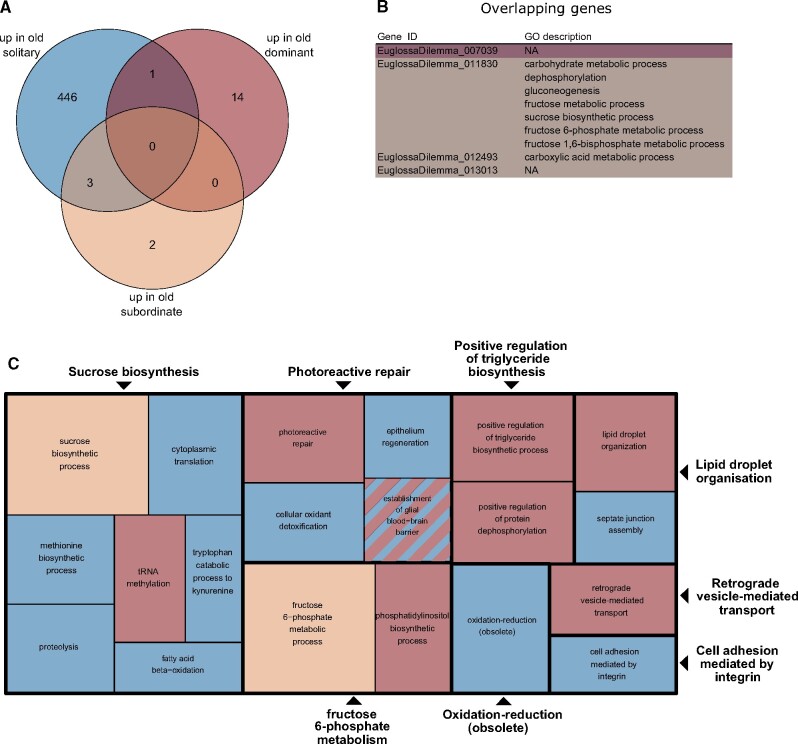
Venn diagram (*A*) and gene ontology (GO) annotations (*B* and *C*) for genes upregulated in old solitary, dominant, and subordinate *Euglossa viridissima* females (i.e., genes upregulated with age). Available GO annotations on the level of Biological Process for genes differentially expressed in multiple comparisons are shown in (*B*) (NA, GO annotations not available), with colors corresponding to the different comparisons as in the Venn diagram (dark purple, up in old solitary/dominant; brown, up in old solitary/subordinate). A reduced visualization of enriched GO terms for genes upregulated in the different comparisons is shown in (*C*), with GO terms clustered based on semantic similarity. Comparisons are color-coded as in (*A*). Each box represents a semantic cluster, and the size of each box is inversely proportional to the adjusted *P* value for the enrichment of GO terms within its cluster. Boxes outlined in bold represent “superclusters” of loosely related GO terms. Three singletons are represented in their own boxes, as they were not assigned to any supercluster.

For a summary of the numbers of DEGs for each comparison, see supplementary table S8, Supplementary Material online (results presented here are those for analyses run on “core individuals,” i.e., excluding *E. dilemma*, outlier eug6 and individual eug25 to avoid the confounding effect of nest). Differential gene expression analyses revealed 902 genes to be significantly differentially expressed with age in solitary females, versus only 100 genes differentially expressed with age in dominant females, and 13 in subordinate females (adjusted *P *<* *0.05; supplementary file S15, Supplementary Material online; figs. 3 and 4). Additionally, solitary and dominant females exhibited increasingly divergent expression profiles with age, with 204 DEGs between young solitary and young dominant females, versus 2,553 DEGs between old solitary and old dominant females. Subordinate females from social nests showed gene expression profiles which were intermediate between those of solitary and dominant females, with 39 DEGs between young solitary and young subordinate females, 47 DEGs between old solitary and old subordinate females, 230 DEGs between young subordinate and young dominant females, and 35 DEGs between old subordinate and old dominant females (for a list of DEGs in each comparison, see supplementary file S15, Supplementary Material online). Finally, old dominant females exhibited considerably more differences to young subordinate females compared with young dominant females (1,104 and 100 DEGs, respectively), despite young dominant females in our study having been born as subordinate females which switched to the dominant position upon their mother’s absence from the nest (see Materials and Methods and fig. 1). It should be noted, however, that young dominant females from our study had all spent more time as dominant than as subordinate, which seems to have been reflected in their gene expression profile. This suggests that molecular changes occur in response to the lifestyle switch, rather than reflecting the position in which females were born.

Functional enrichment of genes upregulated in young compared with old solitary females revealed that these were mainly related to larval feeding behavior, regulation of transcription and mitosis, transmembrane transport, and central nervous system formation (blue boxes in fig. 3*C*, for a full table of functional enrichment results, see supplementary file S16, Supplementary Material online). Genes upregulated in old compared with young solitary females were mainly involved in oxidation–reduction processes, translation, and proteolysis (blue boxes in fig. 4*C*). Genes upregulated in young versus old dominant females were involved in a range of pathways, including cuticular hydrocarbon biosynthesis, signaling, and fatty-acid transport (red boxes in fig. 3*C*). Genes upregulated in old compared with young dominant females were mainly involved in lipid biosynthesis, cellular transport, and tRNA methylation (red boxes in fig. 4*C*). Finally, genes upregulated in young compared with old subordinate females were linked to cuticular hydrocarbon biosynthesis, egg activation, lipid metabolic processes, and zymogen activation (yellow boxes in fig. 3*C*), and genes upregulated in old versus young subordinate females were related to sucrose and fructose synthesis and metabolism (light brown boxes in fig. 4*C*).

Genes which were commonly up- or downregulated with age in both solitary and subordinate females were involved in sucrose/fructose metabolism, and cuticular hydrocarbon biosynthesis (figs. 3*B* and 4*B*).

By contrasting two models in DESeq2 (with vs. without the interaction between age and social status), we detected 411 genes exhibiting different changes in expression with age according to social status (likelihood-ratio test adjusted *P *<* *0.05, supplementary file S15, Supplementary Material online). Functional enrichment of these genes revealed that they were mainly involved in cell adhesion, signaling, and catabolic processes (supplementary file S16, Supplementary Material online).

### Modules of Coexpressed Genes Correlated with Age

Independent identification of genes for which expression patterns correlate with age was performed using the same gene expression data set, following the WGCNA approach (Langfelder and Horvath 2008). A total of 11,929 genes (79% of the 15,046 genes with a nonzero read count, see Materials and Methods and supplementary file S11, Supplementary Material online) were assigned to 29 modules forming a coexpression network. Correlation of each module’s eigengene with age and social status revealed that three modules of coexpressed genes were significantly associated with age (magenta, red, and black modules), one module was associated with the solitary phenotype regardless of age (blue module), two modules were associated with the dominant phenotype (red and blue modules), and no modules were associated with the subordinate phenotype (supplementary fig. S17, Supplementary Material online).

**Fig. 5:**
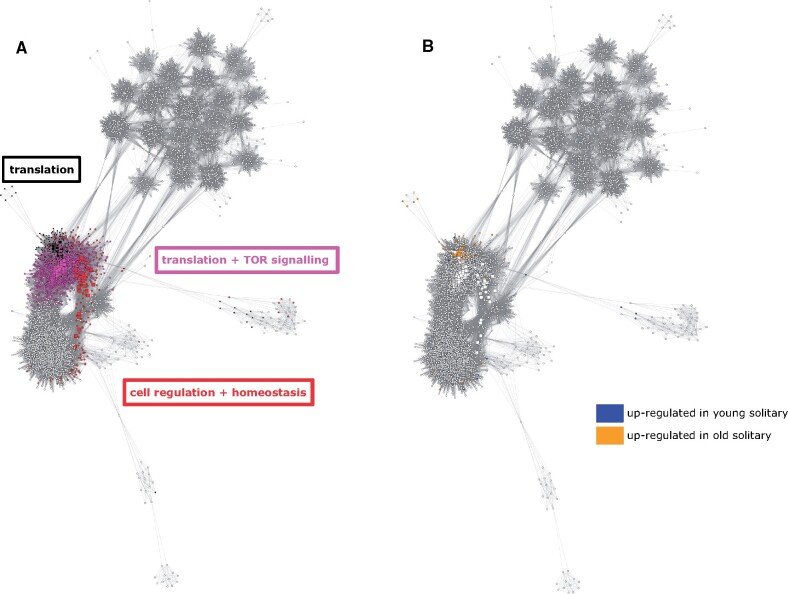
Weighted gene coexpression networks across all expressed genes in *Euglossa viridissima*. Colored nodes in (*A*) represent all genes from the three age-associated modules (black, magenta, red), with each color representing a different module. Square nodes represent hub genes (intramodular connectivity >0.75). Framed words indicate the top functions of the hub (most highly connected) genes in each module. In (*B*), colored nodes represent genes from modules in (*A*) which were differentially expressed between young and old solitary females (blue, upregulated in young; orange, upregulated in old). Colored nodes in network B thus highlight the overlap between the genes found to be significantly associated with age in each independent analysis, that is, the coexpression network analysis and differential gene expression analysis. A reduced version of the network is presented here for visualization purposes, with 6,131 nodes and approximately 600,000 edges.

For the three modules significantly associated with age, we extracted hub genes, that is, genes with an intramodular connectivity >0.75 (intramodular connectivity range 0–1). This resulted in a total of 136 genes (out of 3,939 genes in total belonging to the three age-correlated modules) revealed to be significantly related to age (fig. 5; for the full list of genes, see supplementary file S14, Supplementary Material online, and for functional annotation of the hub genes in each module see supplementary file S16, Supplementary Material online). Functions of hub genes from the three age-related modules were mainly related to translation, TOR signaling, and cell regulation.

**Fig. 6:**
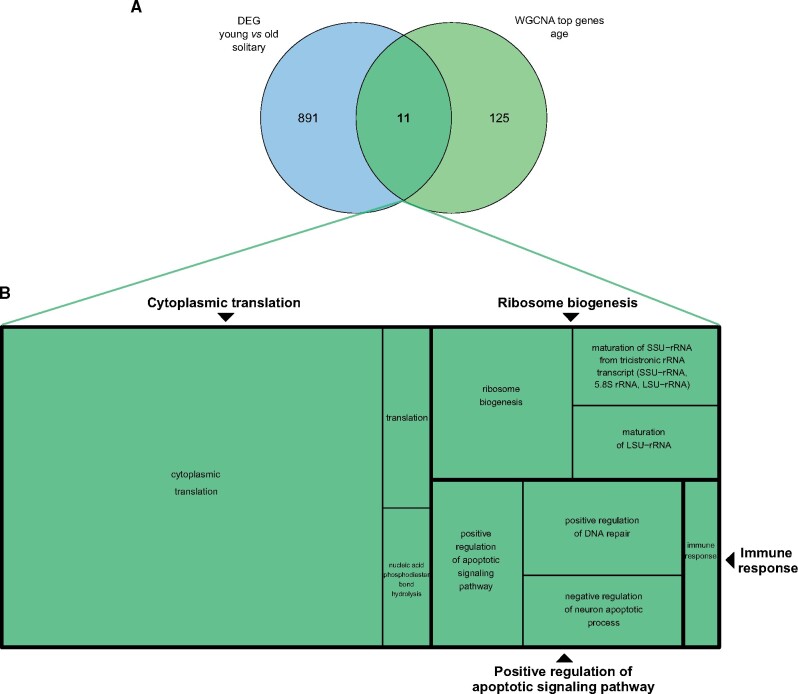
Venn diagram (*A*) and gene ontology (GO) enrichment (*B*) for genes differentially expressed between young and old solitary *Euglossa viridissima* females and that were significantly associated with age in solitary females in the weighted gene coexpression network (WGCNA). The light green circle in (*A*) represents the cumulative 136 hub genes (intramodular connectivity >0.75) across all four age-related modules in the WGCNA. The blue circle shows the number of DEGs between young and old solitary females. A reduced visualization of GO terms which were significantly enriched (adjusted *P* value <0.1) among the 11 genes shown in bold (intersection in the Venn diagram), which were found to be significantly associated with age in solitary females in both the DEG and the WGCN analyses, is shown in (*B*), with GO terms clustered based on semantic similarity. Each box represents a semantic cluster, and the size of each box is inversely proportional to the adjusted *P* value for the enrichment of GO terms within its cluster. Boxes outlined in bold represent “superclusters” of loosely related GO terms. One singleton is represented in its own box, as it was not assigned to any supercluster.

### Overlap of Age-Related Genes between the Differential Gene Expression and the Network Analyses

Of the 136 genes for which expression was significantly correlated with age in the WGCNA, 11 were also found to be significantly differentially expressed between young and old solitary females (figs. 5*B* and 6). Functional enrichment of these overlapping genes revealed that these included genes related to translation, DNA repair/apoptosis, and the immune response. There was no overlap of age-related genes between the two analyses for dominant or subordinate females.

### Known Ageing Genes Differentially Expressed with Age Only in Solitary Females

Of the seven genes known to have a significant impact on longevity in *D. melanogaster*, three genes were differentially expressed with age in solitary *E. viridissima* females (*trachealess, insulin-like 1 receptor*, and *methuselah*, supplementary table S18, Supplementary Material online), whereas none of these genes were differently expressed with age in social females (neither dominant nor subordinate).

### No Change in JH Titers with Age or Social Status

After controlling for nest of origin and sampling date, there were no significant differences in JH titer in relation to age, social status, or the interaction between the two factors (ANOVA, age: χ^2^_(1,21)_ = 0.01, *P *=* *0.92; social status: χ^2^_(2,21)_ = 4.75, *P *=* *0.09; age × social status: χ^2^_(1,21)_ = 0.68, *P *=* *0.41; supplementary figs. S19 and S20, Supplementary Material online). It should however be noted that, with a total sample size of 21 individuals, our statistical power to detect a significant effect was very low (25% for the effect of social status and 7% for the effect of age), therefore these results should be interpreted with caution.

## Discussion

Our results suggest that differential gene expression with age strongly depends on social status. Indeed, most age-related DEGs were specific to a social type (solitary, social dominant, or social subordinate), and no genes were commonly up- or downregulated with age across all social types. This goes against the idea of there being universal hallmarks of ageing, and rather supports the reshaping of major ageing pathways with sociality (Monroy Kuhn and Korb 2016).

The difference in gene expression profiles of young and old solitary females, and the high number of DEGs between these two groups (902 genes) strongly suggest that solitary females undergo substantial physiological changes with age. Functional enrichment of these 902 DEGs revealed that genes upregulated in young solitary females were related to regulation of transcription to an extent higher than expected by chance (i.e., they exhibited significant enrichment for this function), whereas genes upregulated in old solitary females were significantly enriched for terms related to oxidative stress (e.g., fatty acid beta-oxidation, cellular oxidant detoxification). Regulation of transcription has been reported to change with age as certain transcription factors show age-dependent activity (Roy et al. 2002). Interestingly, many of these age-dependent transcription factors are subject to functional alterations by reactive oxygen species, thus oxidative stress may be a factor in the deregulation of transcription with age (Roy et al. 2002). This may explain why we observed lower expression of genes related to the regulation of transcription in old compared with young solitary females, along with an upregulation with age of genes linked to oxidative stress pathways and protein metabolism (translation, proteolysis). The relationship between oxidative stress and ageing has long been suspected, as the accumulation of reactive oxygen species, an inevitable by-product of metabolic activity, is thought to accelerate senescence, therefore making it a potential hallmark of biological ageing (Alonso-Alvarez et al. 2004; Monaghan 2014). The *Drosophila* mutant line *methuselah* and mutants of the *age-1* gene in *C. elegans* are both longer-lived and more resistant to oxidative stress (Larsen 1993; Lin et al. 1998), reinforcing the idea of oxidative stress as a proximate cause of senescence. We found that the *methuselah* homolog in *E. viridissima* (EuglossaDilemma_008076) was upregulated with age in solitary females, perhaps as a response to higher levels of oxidative stress. Alternatively, this expression pattern could be viewed as a downregulation of *methusaleh* in young solitary females, which may in turn explain the upregulation oxidative stress pathways with age.

Another pathway whose expression was found to vary with age more than expected by chance in solitary females was somatic repair (e.g., epithelium regeneration and septate junction assembly). Interestingly, these functions were upregulated with age, suggesting that solitary females invest more in somatic repair as they get older. This may reflect the shift in behavior which solitary females undergo with age in this species, as young foundress females may invest more in reproduction and metabolically costly tasks related to nest construction, whereas older solitary females are primarily guarding their brood inside the nest (Saleh and Ramírez 2019) and may therefore be able to invest more in somatic maintenance.

Although fewer genes were differentially expressed with age in social compared with solitary females, those may provide insight into ageing patterns associated with the social phenotype in *E. viridissima*.

In social dominant females, biological pathways represented more than expected by chance among the genes differentially expressed with age included signaling functions (e.g., netrin-activated signaling pathway, sensory perception of sound) and sodium homeostasis (e.g., response to salt stress, sodium ion export). Perhaps most interestingly, acetyl-CoA biosynthesis was upregulated in young compared with old dominant females and has been proposed as a factor to increase longevity (Shimazu et al. 2010). In humans, higher levels of acetyl-CoA are linked to reduced ageing in the brain (Currais et al. 2019). In old dominant females, functions linked to regulation of gene expression were overrepresented (tRNA methylation, positive regulation of protein dephosphorylation), which may indicate that social dominant females do not experience deregulation of gene expression with age, a phenomenon that has been proposed in many organisms as a hallmark of ageing (Frenk and Houseley 2018).

In social subordinate females, surprisingly, functions related to egg production were upregulated in young individuals. Subordinate females of this species, unlike sterile workers of highly eusocial species, have been reported to lay eggs, although the dominant female systematically replaces subordinate eggs with her own (Saleh and Ramírez 2019). Other pathways which were enriched among age-related genes in subordinate females were mainly linked to metabolism (e.g., lipid metabolic process, fructose 6-phosphate metabolic process), which may reflect the metabolically costly behaviors carried out by this caste (foraging and nest defense, Boff et al. 2015).

The considerable age-related differences in gene expression observed in this study in the solitary *E. viridissima* females but not in the social (neither dominant nor subordinate) females suggest that solitary females undergo more extensive physiological changes with age, particularly when compared with dominant females in social nests. The fact that genes with known links to longevity in *D. melanogaster* were differentially expressed with age in solitary, but not in social, *E. viridissima* females further supports the concept of a remolding of ageing pathways at an initial stage of sociality, even in a facultative eusocial species. That solitary and social dominant females show such marked divergence in their age-related gene expression patterns despite being comparable in terms of age groups is remarkable and supports the idea of coevolution for extended longevity and eusociality already in the early stages of social complexity (Carey 2001). Further studies on other molecular markers related to ageing (e.g., López-Otín et al. 2013) in species along the sociality gradient may help deepen our understanding of this issue.

As subordinate females perform costly tasks such as brood provisioning, and given that workers of obligate eusocial species exhibit considerably shorter lifespan than their queen counterparts (Carey 2001), the absence of major changes in gene expression with age in subordinate *E. viridissima* is somewhat surprising. However, the five genes that were differentially expressed with age in both solitary and subordinate females (figs. 3*A* and 4*A*) provide some insight into the costs of the tasks performed by both types of female in *E. viridissima*. These genes were significantly enriched for functions related to sugar metabolism and the synthesis of cuticular hydrocarbons, which may be related to functions such as protection against desiccation and chemical communication (Drijfhout et al. 2009). Experimental studies in which putatively costly behaviors, such as brood provisioning, are manipulated may further our understanding of the actual costs of such tasks.

Due to the ecology of *E. viridissima*, our study presents some limitations which may have played a role in the differences in gene expression with age observed in the solitary, but not in the social females. First, these may be explained by a potential shift in behavior with age in the solitary females. It has been proposed for the sister species *E. dilemma* that transcriptomic shifts throughout the solitary phases of the life cycle may be attributed to a shift in behavior, as the female transits from an actively foraging foundress to a relatively inactive guard, caring for her brood (Saleh and Ramírez 2019). Similar to the differences observed between young and old solitary females in our study species *E. viridissima*, Saleh and Ramírez (2019) found that metabolic pathways were upregulated in foundresses compared with females in the guarding phase. Additional information on the life cycle and associated shifts in behavior in *E. viridissima* would, therefore, allow us to better account for this potential factor, and determine whether the differences observed in our study are due to ageing per se as opposed to behavioral repertoire expressed at the time of collection*.* Another factor that may explain the lack of ageing signs in dominant females is oophagy. Video recordings of *E. dilemma* nests revealed that oophagy, whereby the dominant female ingests eggs laid by subordinate females in her nest to replace them with her own, occurs commonly in this species (Saleh and Ramírez 2019). If this behavior also occurs in the closely related *E. viridissima*, this could constitute a notable nutritional source for dominant females, thereby reducing any trade-off generated by the allocation of limited resources. Thirdly, due to the life cycle of the species, young dominant females could only be sampled after an old dominant female had left the nest and a subordinate had taken its place as dominant, requiring behavioral observations before sampling. Thus, young dominant females could not be sampled as quickly after the first observation compared with young solitary females, making the age gap between young and old dominant females smaller than for solitary females. This limitation is difficult to overcome in this species, but similar studies in other systems where it is possible to sample younger dominant females would help clarify to what extent this affected the gene expression patterns in our study.

Our gene coexpression network analysis was performed in part to identify age-related genes overlapping with those differentially expressed with age, thus narrowing down the list of genes that potentially play a role in the ageing process. The results from the coexpression network analysis highlighted modules of genes for which expression was correlated with age, independent of social status. Functional enrichment of hub genes from these modules revealed that they were linked to well-established age-related pathways to an extent greater than expected by chance, for example, the TOR signaling pathway (Kapahi et al. 2004; Partridge et al. 2011). TOR acts as a key regulator of protein translation in response to dietary cues (Kennedy and Kaeberlein 2009) and fittingly, we found that hub genes from age-related modules were also enriched for functions linked to translation as well as cell regulation and homeostasis in *E. viridissima*, which have similarly been proposed as hallmarks of cellular ageing (López-Otín 2013; Frenk and Houseley 2018). Thus, hub genes from age-related modules in the coexpression network presented here may help to disentangle the effects of ageing versus the effects of a shift in behavior, especially within solitary nests.

The 11 genes consistently related to ageing in both our DEG and WGCN analyses represent particularly good candidates for further functional tests (e.g., knock-down experiments) to investigate their role in ageing processes. Two major processes were represented more than expected by chance among these overlapping genes: RNA translation and DNA damage repair/apoptosis. Ribosome biogenesis and activity have been proposed to play a central role in ageing processes (Buchwalter and Hetzer 2017; Gonskikh and Polacek 2017), as the regulation of translation has an impact on cellular proteostasis, and hence lifespan (Steffen and Dillin 2016). Pathways which respond to DNA damage in the cell, either through repair of the damages or apoptosis, also have reported links to ageing. Defects in the DNA repair machinery can lead to premature ageing (Lombard et al. 2005), as can defects in the apoptosis pathway since apoptosis is important for eliminating damaged cells which arise due to oxidative stress or DNA damage (Higami and Shimokawa 2000).

We report here the first data on JH titer levels in an orchid bee species. We found no significant difference in the hemolymph JH titers between young versus old and dominant versus subordinate females (supplementary fig. S19, Supplementary Material online). Yet, despite a caveat in the interpretation of these data due to the low sample number for solitary individuals (supplementary fig. S20, Supplementary Material online) and hence the low statistical power, some conclusions are possible. First of all, these findings stand in apparent contrast to the marked increase in JH levels seen in honeybee workers as they age and transit from nursing to foraging tasks (Huang and Robinson 1996), as well as the equally strong positive correlation in JH levels with social status in the bumblebee *B. terrestris* (Bloch et al. 2000; Shpigler et al. 2014). In the stingless bee *Melipona quadrifasciata*, however, JH hemolymph titers in old workers (foragers) are lower than those in younger ones (Cardoso-Júnior et al. 2017). These evident discrepancies in the role of JH among representatives of the three social tribes of the monophyletic corbiculate bees, and now also a facultatively social euglossine, indicate that in corbiculate bees, which is the only clade within the bees that comprises members of all social complexities, there is no strong link between JH and reproduction, that is, social status. Rather, each tribe appears to have remolded this ancient gonadotropic insect hormone (Santos et al. 2019) according to its idiosyncratic mode of social biology. Such idiosyncrasies are not unique to the corbiculate bees, but are also found in wasps. For instance, in the solitary progressively provisioning eumenine wasp *S. cornuta*, there is also no correlation between JH levels and age or reproductive status (Kelstrup et al. 2018). Further, two sympatric species of the same genus of *Polistes* paper wasps showed divergent patterns of JH titer with respect to age/task and social status (Kelstrup et al. 2015). Hence, the lack of a link between JH and age or social status in *E. viridissima* is not such a surprising result. Nevertheless, recent data based on a WGCNA of *B. terrestris* workers with experimentally manipulated JH levels (allatectomy and JH replacement) indicate a cost imposed by JH on brain and fat body gene expression (Shpigler et al. 2020). The loss of the gonadotropic function of JH in the highly eusocial honeybee could be one mechanism by which its long-lived queens avoid disruption to brain and fat body gene expression.

To conclude, the extensive changes in gene expression with age in solitary *Euglossa* females, which stand in stark contrast to the absence of gene expression changes with age in social *Euglossa* females, provide insight into the molecular pathways underlying ageing in solitary organisms, and how these may be modified in eusocial individuals. Our gene coexpression network further highlighted several hub genes correlated with age that were related to translation, TOR signaling, and cell homeostasis. The overlap of genes for which expression levels are associated with ageing across differential expression and coexpression network analyses revealed that a subset of genes related to translation, DNA repair, and apoptosis represent particularly relevant candidates for future ageing studies, both in solitary and social individuals. Ultimately, these results in a socially polymorphic species represent a tentative step in understanding the genetic mechanisms allowing the reversal of the fecundity/longevity trade-off which seemingly accompanies the evolutionary transition to eusociality (Carey 2001). Our study, based on expression differences inferred from transcriptome data, represents a useful starting point in the search for genetic markers and proximate mechanisms of ageing in the transition from solitary life to eusociality. A necessary next step in future studies would be to experimentally test the function of candidate genes identified here in order to eliminate possible confounding factors such as shifts in behavior with age, for instance through knock-down experiments to corroborate or refute the role of these genes in relation to senescence.

Finally, in order to determine the relevance of these genes in the context of the fecundity/longevity trade-off, additional life history data are needed, such as information on the lifespan and reproductive output of individuals, to determine how these relate to expression of the candidate genes. Due to the invasive nature of RNA sampling from such small organisms, this is difficult to achieve for *E. viridissima*. Emerging technologies may make this an accessible goal in the future, or studies in species for which larger sample sizes are available may allow parallel sampling of a subset of individuals alongside life history observations in the same or similar individuals.

## Supplementary Material

Supplementary data are available at *Genome Biology and Evolution* online.

## Supplementary Material

evab075_Supplementary_Data
