# Supplementary material for: Transcriptomic Signatures of Ageing Vary in Solitary and Social Forms of an Orchid Bee
Source: Genome Biol Evol. 2021 Apr 29;13(6):evab075. doi: 10.1093/gbe/evab075 (PMC8214409; doi:10.1093/gbe/evab075)
Supplement: evab075_Supplementary_Data [file evab075_supplementary_data.zip › Supplementary_material_graphs_and_text.pdf]

Séguret *et al*. Transcriptomic signatures of ageing vary in solitary and social forms of an orchid bee

Supplementary material – graphs and text

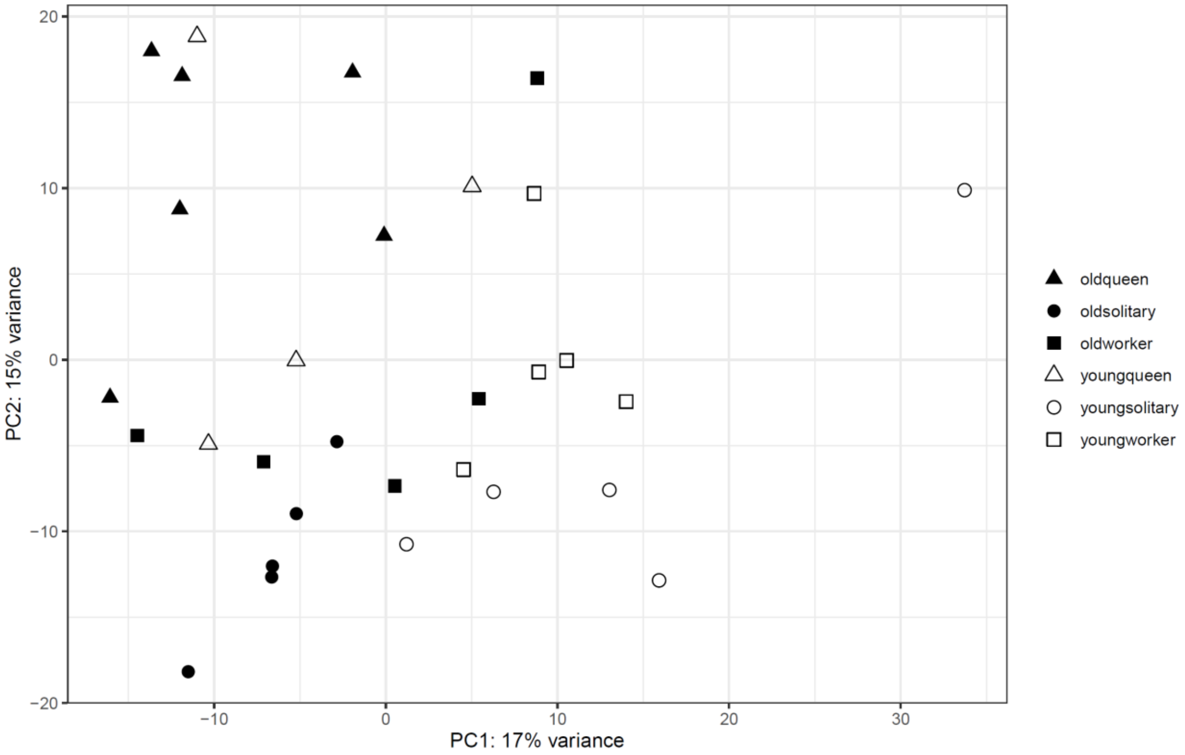

Fig. S4. Principal component analysis (PCA) of variance-stabilised RNA read counts for young and old females, from solitary and social nests. All individuals sequenced, including *E. dilemma* individuals and the one outlier (young solitary, top right), are represented in this figure. Each point represents the expression profile across all genes for one individual. Axis labels indicate the amount of variance in gene expression explained by the first two principal components (PC1 and PC2).

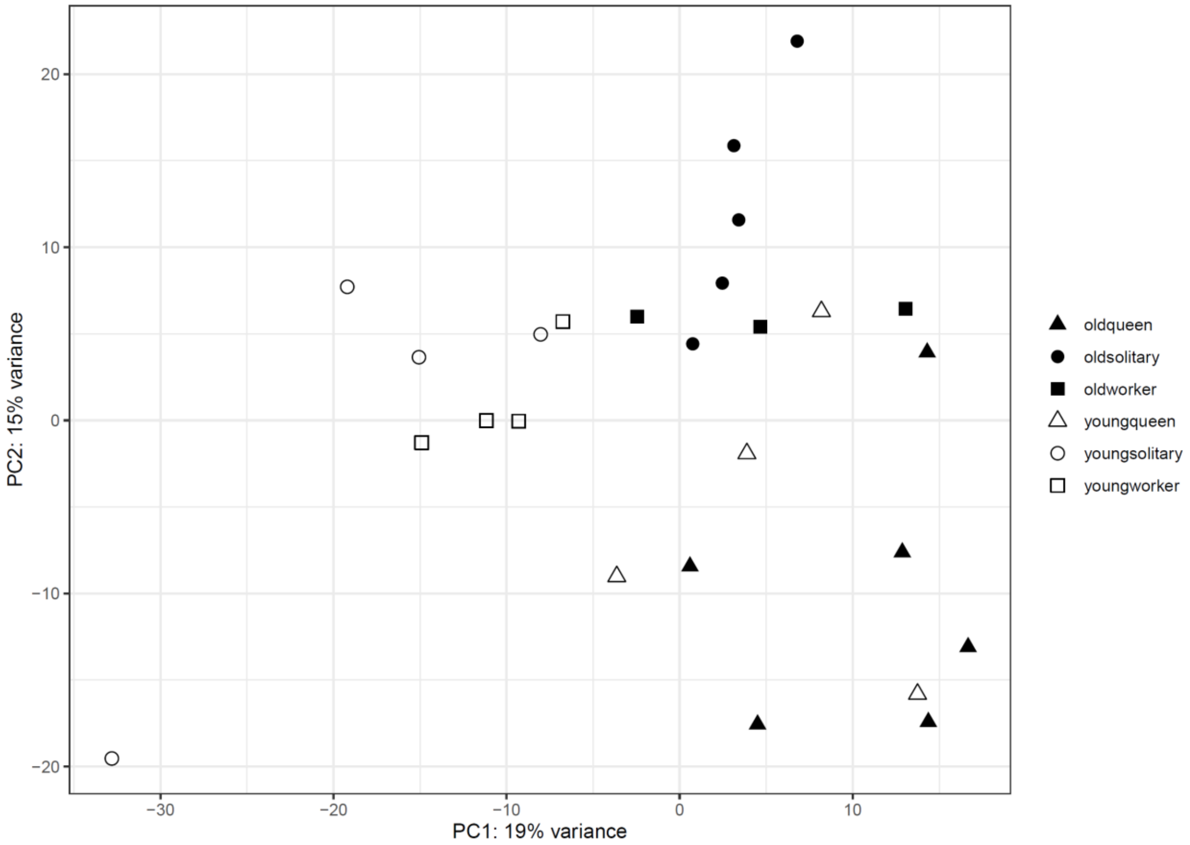

Fig. S5. Principal component analysis (PCA) of variance-stabilised RNA read counts for young and old females, from solitary and social nests. All *E. viridissima* individuals are represented here, including the one outlier (young solitary, bottom left). Each point represents the expression profile across all genes for one individual. Axis labels indicate the amount of variance in gene expression explained by the first two principal components (PC1 and PC2).

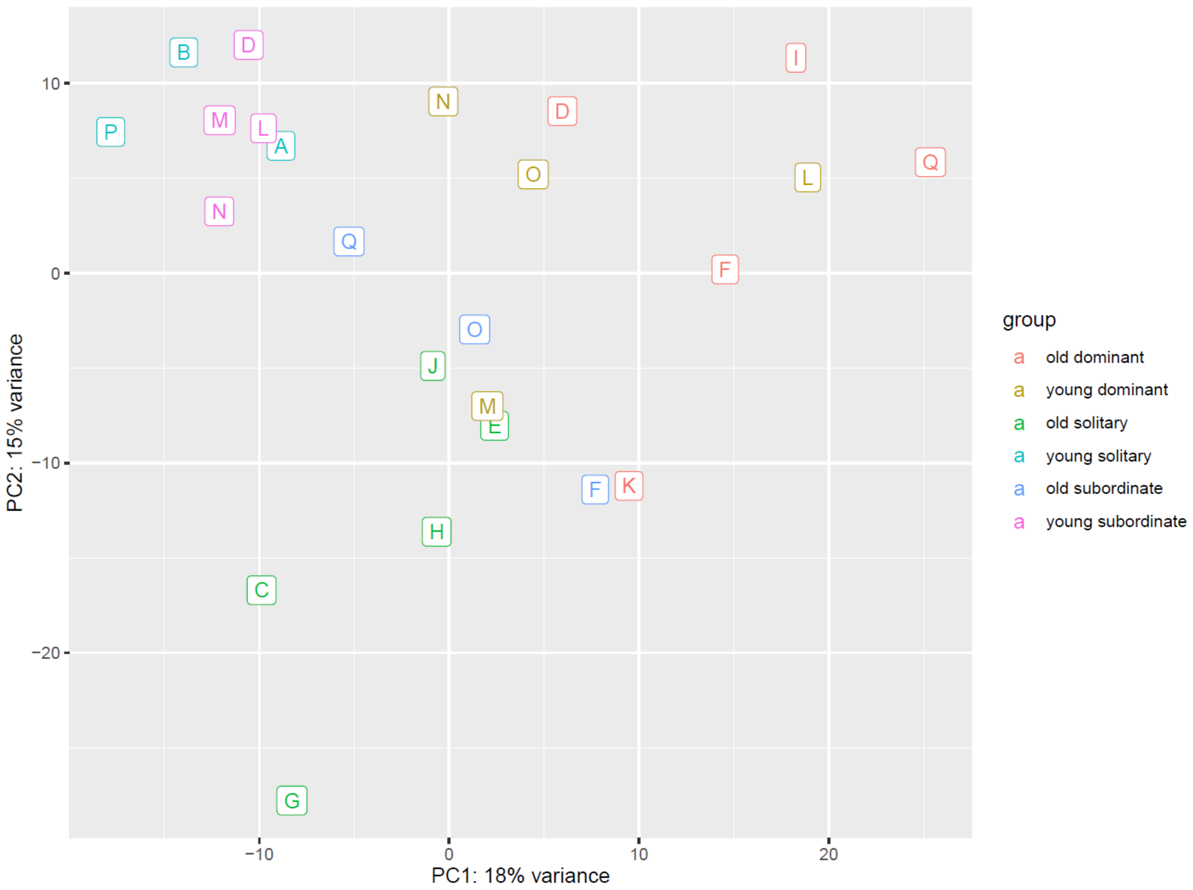

Fig. S6. Principal component analysis (PCA) of variance-stabilised RNA read counts for young and old females, from solitary and social nests. Only “core individuals” are represented here (*E. viridissima* individuals excluding outlier eug6 and individual eug25 to avoid confounding effect of nest). Each point represents the expression profile across all genes for one individual. Letters on the graph represent the individual’s nest of origin. Axis labels indicate the amount of variance in gene expression explained by the first two principal components (PC1 and PC2).

File S9. Detailed description of the pipeline for transcriptome assembly and *E. dilemma* genome annotation Edil_v3.0.

The transcriptomic analyses are based on the previously published draft genome sequence assembly for *E. dilemma* (GCA_002201625.1, (Brand et al. 2017)). Repeats were softmasked (35.30% of the total genome assembly length) using bedtools (v2.27.1) based on repeat annotations from Tandem Repeats Finder (v4.09, (Benson 1999)) and RepeatMasker (Smit, AFA, Hubley, R & Green, P. 2013-2020). To improve the previous annotation of this genome, which was based solely on gene predictions and homology to *Apis mellifera* proteins (Brand et al. 2017), we used funannotate (v1.8.1, Palmer & Stajich 2020) with the previous gene annotation (edil.1.0.annotations.gff, (Brand et al. 2017)), novel experimental evidence derived from RNA-seq data (generated in this study: RNA-seq paired-end reads (euglossa.R[1,2].fastq.gz) aligned to the *E. dilemma* genome with HiSat2 (v2.1.0, (Kim, Langmead, and Salzberg 2015)) in conjunction with processing with samtools (v1.8, (Li et al. 2009)) and sambamba (v0.7.1, (Tarasov et al. 2015)) (euglossa.all.hisat2.bam), transcriptome assembly with binpacker (v1.1, (Liu et al. 2016)) from 4 samples (binpacker.transcriptome.fa), genome-guided transcriptome assembly from one sample (eug33, stringtie2.transcriptome.gtf) with stringtie2 (v1.3.3b, (M. Pertea et al. 2016))), a RNA-seq data-based Trinity (v1.5.1, (Grabherr et al. 2011)) transcriptome assembly of the closely related *E. dilemma* (euglossa.dilemma.trinity.transcriptome.fa, P. Brand, pers. comm., data published in Brand et al. 2020).. Further, protein sequences from 11 related bee species (*Bombus impatiens*: GCF_000188095.2 BIMP_2.1_protein.faa; *B. terrestris*: GCF_000214255.1 Bter_1.0_protein.faa; *Apis mellifera*: GCF_003254395.2 Amel_HAv3.1_protein.faa; *Melipona quadrifasciata*: GCA_001276565.1 ASM127656v1_protein.faa; *Eufrisea mexicana*: GCF_001483705.1 ASM148370v1_protein.faa, *Friseomelitta varia* GCA_011392965.1_Fvar_1.2_protein.faa, *Megachile rotundata* GCF_000220905.1_MROT_1.0_protein.faa, *Habropoda laboriosa* GCF_001263275.1_ASM126327v1_Habropoda_protein.faa, *Dufourea novaeabgliae* GCF_001272555.1_ASM127255v1_Dufourea_protein.faa, *Megalopta genalis* GCF_011865705.1_USU_MGEN_1.2_protein.faa, *Nomia melanderi* GCF_003710045.1_USU_Nmel_1.2_protein.faa, (Wallberg et al. 2019; Kapheim et al. 2015, Kapheim et al. 2020, Kapheim et al. 2019)), and Uniprot (sprot) was used for homology-based evidence.

In brief, PASA (v2.4.1, (B. J. Haas et al. 2008)) was used to train gene predictions (funannotate train --cpus $CPUs --species "$SPECIES" --max_intronlen 10000 --pasa_alignment_overlap 30.0 --pasa_db sqlite --no_trimmomatic --coverage 50 --min_coverage 5 --memory 100G --stranded no --aligners minimap2 --pasa_min_pct_aligned 85 --pasa_min_avg_per_id 90 --pasa_num_bp_splice 3 --trinity euglossa.dilemma.trinity.transcriptome.fa --input $ASM --out $OUT --left euglossa.R1.fastq.gz --right euglossa.R2.fastq.gz).

With this training set, funannotate (v1.8.1) prediction was performed (funannotate predict -i $ASM -o $OUT -s "$SPECIES" --name $NAME"_" --weights augustus:2 pasa:6 codingquarry:1 --rna_bam $BAMFILE --busco_seed_species bombus_impatiens1 --busco_db hymenoptera --cpus $CPUs --keep_no_stops --keep_evm --SeqCenter XXXX --organism other --max_intronlen 10000 --ploidy 1 --repeat_filter overlap blast --soft_mask 5000 --protein_evidence $INPUT2 $INPUT4 $INPUT5 $INPUT6 $INPUT7 $INPUT8 $INPUT9 $INPUT10 $INPUT11 $INPUT12 $INPUT13 $INPUT14 --stringtie $STRINGTIE --transcript_evidence $BINPACKER Edil_v1.0_transcripts.fa --other_gff edil.1.0.annotations.formatted.fixed_clean.gff:1) . In this step transcriptomes (Edil1.0) and proteomes (11 bee species, see above) are aligned to the *E. dilemma* genome with minimap2 (v2.17-r941, (Li 2018)) and proteins aligned with Diamond (v2.0.444, (Buchfink, Xie, and Huson 2015)) and exonerate (v2.4.0, (Slater and Birney 2005)), gene predictions are generated based de novo on the genome sequence by Genemark-ES (n=31318 gene models , v4.35, (Borodovsky and Lomsadze

2011)), snap (with PASA training set of 7137 gene models: n=89370, v2006-07-28, Korf 2004), glimmerhmm (with PASA training set of 7137 gene models: n=48791, v3.0.4, Majoros et al. 2004) and Augustus (with PASA training set of 7137 gene models: n=8579 models of which n=6480 were high quality, v3.3.3, (Hoff and Stanke 2019)), based on RNA-seq alignments by CodingQuarry (n=21826 gene models, v2.0, (Testa et al. 2015)), gene models from PASA (n=7137) and gene models from the previous annotation (Edil1.0, Brand et al., n=15904), and then integrated in Evidence Modeler (v.1.1.1, (B. J. Haas et al. 2008)) to a total of 22,039 gene models. Too short (< 50 aa, n=27), gap-spanning (n=49) or repeat-overlapping (n=4539) gene models were removed so that 17,424 protein-coding gene models remained. Additionally, 145 tRNA gene models were found with tRNAscan-SE (v2.0.6, (Chan and Lowe 2019)), so that the total of predicted gene-model was n= 17,569.

Gene models are then updated with PASA and transdecoder (v5.5.0, Haas and Papanicolaou (B. J. A. P. Haas 2019)) (funannotate update -i $OUT --cpus $CPUs --left $FWD --right $REV --memory 100G --stranded no --pasa_db sqlite --species "$SPECIES" --max_intronlen 10000 --pasa_alignment_overlap 30.0 --coverage 50 --trinity $TRINITY), using empirical evidence (RNA-seq reads: euglossa.R[1,2].fastq.gz, Trinity transcriptome assembly: euglossa.dilemma.trinity.transcriptome.fa). For 17,424 protein-coding gene models and PASA aligned transcripts, Kallisto (v0.46.1) TPM value were used to update gene models and to determine which PASA gene models to select at each locus. One problematic gene models was manually fixed (funannotate fix). This yielded 17,550 protein-coding genes and 144 t-RNAs.

Then genes were functionally annotated (funannotate iprscan --method local --cpus 110 --num 1000 --out interproscan.output.xml; funannotate annotate --busco_db hymenoptera, --iprscan interproscan.output.xml), including HMMer (v2.3.2, v3.3.1b2, (Mistry et al. 2013)), Diamond blastp searches of PFAM version 33.1 ((El-Gebali et al. 2019)), UniProt DB version 2018_11, EggNog Annotations (eggnog mapper v1.0.3 (Huerta-Cepas et al. 2017), eggnog_4.5/hmmdb databases: arthopoda, insecta, hymanoptera, drosophila, database created with "download_eggnog_data.py euk artNOG inNOG meNOG droNOG hymNOG" and "diamond getseq --db eggnog_proteins_old.dmnd | diamond makedb --db eggnog_proteins.dmnd"), Diamond blastp search of MEROPS version 12.0 (Rawlings et al. 2018), annotating CAZYmes using HMMer search of dbCAN version 7.0 (Huang et al. 2018), annotating proteins with BUSCO hymenoptera models (v3.0.2, Hymenoptera odb9, (Simão et al. 2015)), predicting secreted proteins with SignalP (4.1, (Nielsen 2017)), InterProScan5 annotations (v81.0, (Jones et al. 2014)). In total, we added 102,019 functional annotations to the gene models.

The resulting final set of 17,550 protein coding genes from 17,528 protein coding loci and 144 tRNA genes were, checked for completeness using BUSCO4 (v4.1.4, (Simão et al. 2015), command: busco -i $INPUTFILE --force --mode proteins --lineage_dataset $LINEAGE --offline --augustus_species $AUGUSTUSSPECIES --cpu $CPUs --evalue 1e-03 --limit 10 --out $INPUTFILENAME.busco4), showing an increase in completeness compare to the previous annotation (Edil1.0: C:61.8%[S:42.8%,D:19.0%],F:10.7%,M:27.5%,n:5991; this annotation (proteins): C:86.5%[S:84.8%,D:1.7%],F:6.7%,M:6.8%,n:5991; this annotation (cds-transcripts): C:85.2%[S:83.6%,D:1.6%],F:5.8%,M:9.0%,n:5991, this annotation (mRNA-transcripts): C:85.3%[S:83.7%,D:1.6%],F:5.7%,M:9.0%,n:5991), but have not reached the completeness (of BUSCO genes) found in the genome, whether repeats were masked (C:92.6%[S:91.1%,D:1.5%],F:3.7%,M:3.7%,n:5991) or unmasked (C:92.6%[S:91.1%,D:1.5%],F:3.7%,M:3.7%,n:5991).

The final annotation totalling 17,550 protein coding models from 17,528 loci and 144 t-RNA gene models was deposited in Dryad, doi:10.5061/dryad.2547d7wnh.

To facilitate functional enrichment, we additionally retrieved GO terms from Biomart Ensembl databases through modified scripts from Colgan et al. (2019) and the R package biomaRt (Durinck et al. 2009), using *Apis mellifera* (HVa3.1) orthologs to our annotated *E. dilemma* proteins, generated by broccoli (Derelle et al. 2020). For each ortholog found in *Apis mellifera*, GO terms were retrieved from biomart Ensembl and amended with GO terms from existing *Drosophila orthologs* for the *Apis mellifera orthologs*. These GO terms, merged with GO terms assigned through the funannotate pipeline were then used for enrichment analyses (n=8530 transcripts with GO terms).

References

Benson G. 1999. Tandem Repeats Finder: a program to analyze DNA sequences. Nucleic Acids Res. 27(2): 573-580. https://doi.org/10.1093/nar/27.2.573.

Borodovsky M, Lomsadze A. 2011. Eukaryotic gene prediction using GeneMark.hmm-E and GeneMark-ES. Curr Protoc Bioinformatics. 35(1): 4.6.1-4.6.10. https://doi.org/10.1002/0471250953.bi0406s35.

Brand P, Saleh N, Pan H, Li C, Kapheim KM, Ramírez SR. 2017. The nuclear and mitochondrial genomes of the facultatively eusocial orchid bee *Euglossa dilemma*. G3 (Bethesda). 7(9): 2891-2898. https://doi.org/10.1101/123687.

Buchfink B, Xie C, Huson DH. 2015. Fast and sensitive protein alignment using DIAMOND. Nat Methods. 12(1): 59–60.

Chan PP, Lowe TM. 2019. tRNAscan-SE: searching for tRNA genes in genomic sequences. Methods Mol Biol. 1962: 1–14.

Colgan TJ, Fletcher IK, Arce AN, Gill RJ, Ramos Rodrigues A, Stolle E, Chittka L, Wurm Y. 2019. Caste- and pesticide-specific effects of neonicotinoid pesticide exposure on gene expression in bumblebees. Mol Ecol. 28(8): 1964-1974.

Derelle R, Philippe H, Colbourne JK. 2020. Broccoli: Combining phylogenetic and network analyses for orthology assignment. Mol Biol Evol. 37(11): 3389–3396.

Durinck S, Spellman P, Birney E, Huber W. 2009. Mapping identifiers for the integration of genomic datasets with the R/Bioconductor package biomaRt. Nat Protoc. 4: 1184–1191.

El-Gebali S, Mistry J, Bateman A, Eddy SR, Luciani A, Potter SC, Qureshi M, Richardson LJ, Salazar GA, Smart A, Sonnhammer ELL. 2019. The Pfam protein families database in 2019. Nucleic Acids Res. 47(D1): D427–32.

Grabherr MG, Haas BJ, Yassour M, Levin JZ, Thompson DA, Amit I, Adiconis X, Fan L, Raychowdhury R, Zeng Q, Chen Z, Mauceli E, Hacohen N, Gnirke A, Rhind N, di Palma F, Birren BW, Nusbaum C, Lindblad-Toh K, Friedman N, Regev A. 2011. Full-length transcriptome assembly from RNA-Seq data without a reference genome. Nat Biotechnol. 29(7):644-52. doi: 10.1038/nbt.1883.

Haas BJ, Papanicolaou A. 2019. *TransDecoder* (version v5.5.0). https://github.com/TransDecoder/TransDecoder/wiki.

Haas BJ, Salzberg SL, Zhu W, Pertea M, Allen JE, Orvis J, White O, Buell CR, Wortman JR. 2008. Automated eukaryotic gene structure annotation using EVidenceModeler and the program to

assemble spliced alignments. Genome Biol. 9(1): R7.

Hoff KJ, Stanke M. 2019. Predicting genes in single genomes with AUGUSTUS. Curr Protoc Bioinformatics. 65(1): e57.

Huang L, Zhang H, Wu P, Entwistle S, Li X, Yohe T, Yi H, Yang Z, Yin Y. 2018. dbCAN-Seq: A database of carbohydrate-cctive enzyme (CAZyme) sequence and annotation. Nucleic Acids Res. 46(D1): D516–21.

Huerta-Cepas J, Forslund K, Coelho LP, Szklarczyk D, Jensen LJ, von Mering C, Bork P. 2017. Fast genome-wide functional annotation through orthology assignment by eggNOG-Mapper. Mol Biol Evol. 34(8): 2115–22.

Jones P, Binns D, Chang HY, Fraser M, Li W, McAnulla C, McWilliam H, Maslen J, Mitchell A, Nuka G, Pesseat S. 2014. InterProScan 5: genome-scale protein function classification. Bioinformatics. 30(9): 1236–40.

Kapheim KM, Jones BM, Pan H, Li C, Harpur BA, Kent CF, Zayed A, Ioannidis P, Waterhouse RM, Kingwell C, Stolle E, Avalos A, Zhang G, McMillan WO, Wcislo WT. 2020. Developmental plasticity shapes social traits and selection in a facultatively eusocial bee. PNAS. 117 (24) 13615-13625.

Kapheim KM, Pan H, Li C, Blatti C, Harpur BA, Ioannidis P, Jones BM, kent CF, Ruzzante L, Sloofman L, Stolle E, Waterhouse RM, Zayed A, Zhang G, Wcislo WT. 2019. Draft genome assembly and population genetics of an agricultural pollinator, the solitary alkali bee (Halictidae: *Nomia melanderi*). G3. 9(3): 625-634.

Kapheim KM, Pan H, Li C, Salzberg SL, Puiu D, Magoc T, … Zhang G. 2015. Social evolution. Genomic signatures of evolutionary transitions from solitary to group living. Science. 348(6239): 1139–43.

Kim D, Langmead B, Salzberg SL. 2015. HISAT: A fast spliced aligner with low memory requirements. Nat Methods. 12(4): 357–60.

Korf I. 2004. Gene finding in novel genomes. BMC Bioinformatics. 5: 59.

Li H, Handsaker B, Wysoker A, Fennell T, Ruan J, Homer N, Marth G, Abecasis G, Durbin R, 1000 Genome Project Data Processing Subgroup. 2009. The sequence alignment/map format and SAMtools. Bioinformatics. 25(16): 2078–79.

Li H. 2018. Minimap2: pairwise alignment for nucleotide sequences. Bioinformatics. 34(18): 3094-3100. https://doi.org/10.1093/bioinformatics/bty191.

Liu J, Li G, Chang Z, Yu T, Liu B, McMullen R, Chen P, Huang X. 2016. BinPacker: packing-based de novo transcriptome assembly from RNA-seq data. PLoS Comp Biol. 12(2): e1004772.

Majoros WH, Pertea M, Salzberg SL. 2004. TigrScan and GlimmerHMM: two open-source ab initio eukaryotic gene-finders. Bioinformatics. 2878-2879

Mistry J, Finn RD, Eddy SR, Bateman A, Punta M. 2013. Challenges in homology search: HMMER3 and convergent evolution of coiled-coil regions. Nucleic Acids Res. 41(12): e121.

Nielsen H. 2017. Predicting secretory proteins with SignalP. Methods Mol Biol. 1611: 59–73.

Palmer JM, Stajich J. 2020. Funannotate v1.8.1: Eukaryotic genome annotation (Version v1.8.1). Zenodo. http://doi.org/10.5281/zenodo.4054262

Pertea M, Kim D, Pertea GM, Leek JT, Salzberg SL. 2016. Transcript-level expression analysis of RNA-seq experiments with HISAT, StringTie and Ballgown. Nat Protoc. 11(9): 1650–67.

Rawlings ND, Barrett AJ, Thomas PD, Huang X, Bateman A, Finn RD. 2018. The MEROPS database of proteolytic enzymes, their substrates and inhibitors in 2017 and a comparison with peptidases in the PANTHER database. Nucleic Acids Res. 46(D1): D624-D632. https://doi.org/10.1093/nar/gkx1134.

Simão FA, Waterhouse RM, Ioannidis P, Kriventseva EV, Zdobnov EM. 2015. BUSCO: assessing genome assembly and annotation completeness with single-copy orthologs. Bioinformatics. 31(19): 3210–12.

Slater GSC, Birney E. 2005. Automated generation of heuristics for biological sequence comparison. BMC Bioinformatics. 6: 31.

Smit AFA, Hubley R, Green P. 2013-2020. RepeatMasker (version 4.0). http://www.repeatmasker.org.

Tarasov A, Vilella AJ, Cuppen E, Nijman IJ, Prins P. 2015. Sambamba: fast processing of NGS alignment formats. Bioinformatics. 31(12): 2032–34.

Testa AC, Hane JK, Ellwood SR, Oliver RP. 2015. CodingQuarry: highly accurate Hidden Markov Model gene prediction in fungal genomes using RNA-seq transcripts. BMC Genomics. 16: 170.

Wallberg A, Bunikis I, Pettersson OV, Mosbech MB, Childers AK, Evans JD, Mikheyev AS, Robertson HM, Robinson GE, Webster MT. 2019. A hybrid de novo genome assembly of the honeybee, *Apis mellifera*, with chromosome-length scaffolds. BMC Genomics. 20(1): 275.

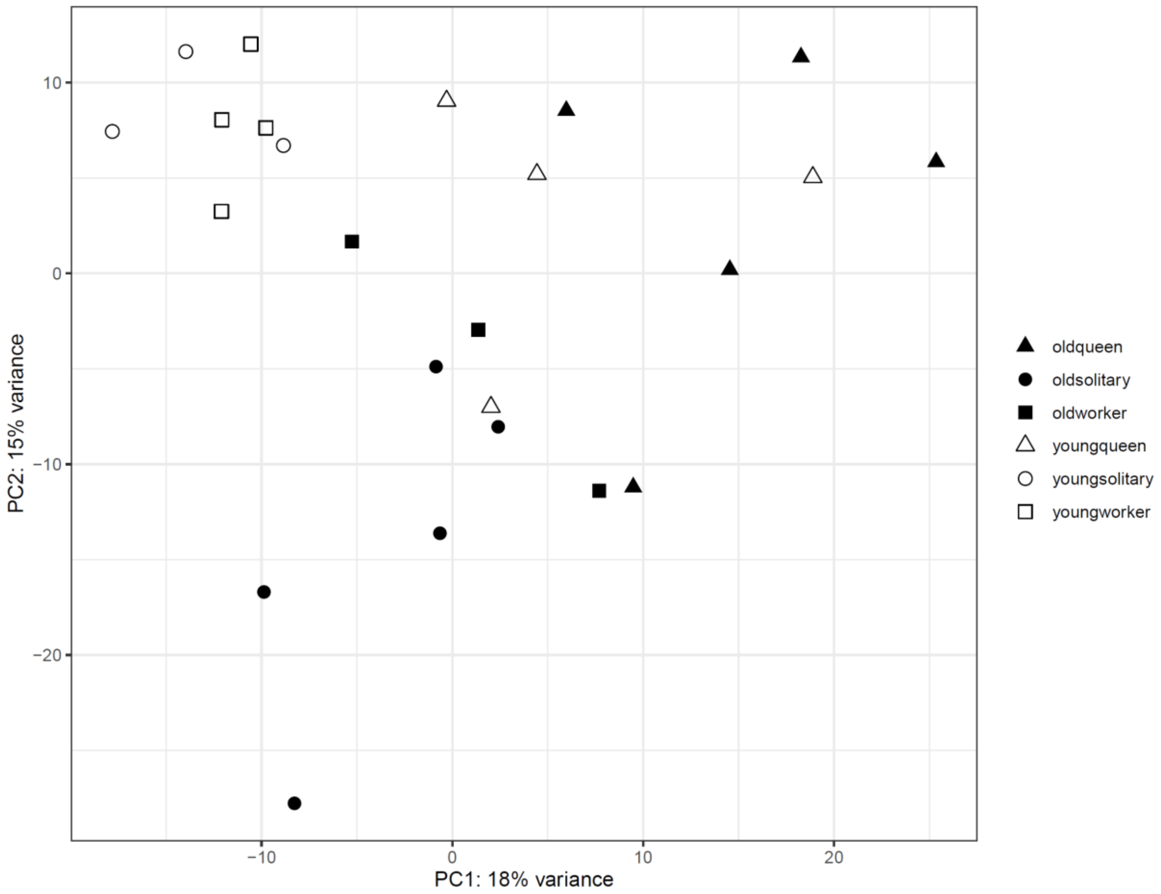

Fig. S13. Principal component analysis (PCA) of variance-stabilised RNA read counts for young and old females, from solitary and social nests. Each point represents the expression profile across all genes for one individual. Only core individuals are represented in this figure, *i.e.* excluding *E. dilemma* individuals, outlier eug6, and individual eug25 to avoid the confounding effect of nest. Axis labels indicate the amount of variance in gene expression explained by the first two principal components (PC1 and PC2).

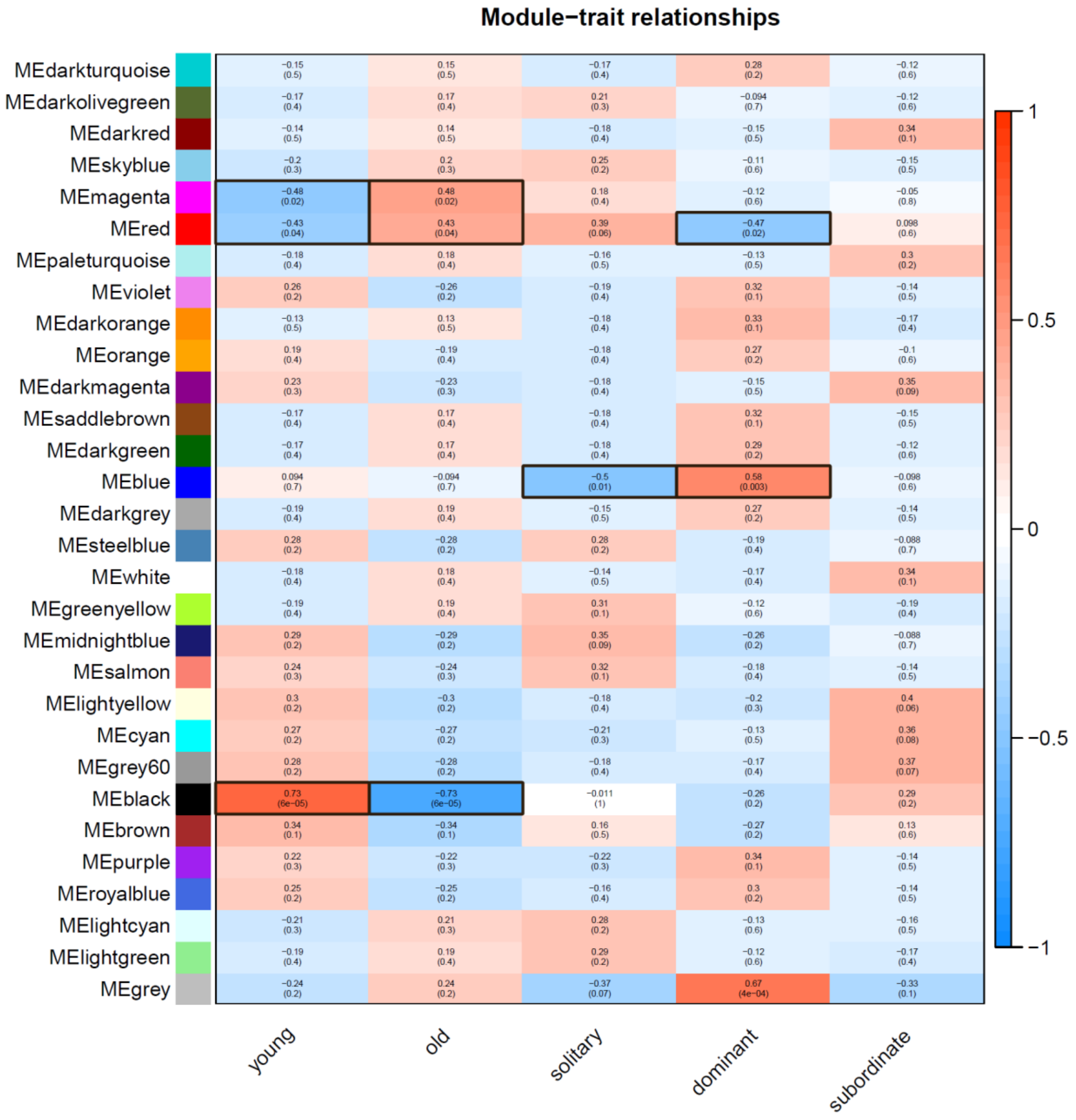

Fig. S17. Heatmap representing the correlations between module eigengenes and external traits of interest: age and social status (subordinate, dominant or solitary). Numbers shown in each square represent the correlation coefficient, with the associated p-value in brackets. Red squares indicate a positive correlation between the module eigengene and the trait; blue squares indicate a negative correlation. Modules with a p-value < 0.05 for a particular trait (highlighted by a black outline) were considered to be significantly correlated with that trait. Note: the grey module consists of unassigned genes and is therefore not interpreted in the relation to a trait.

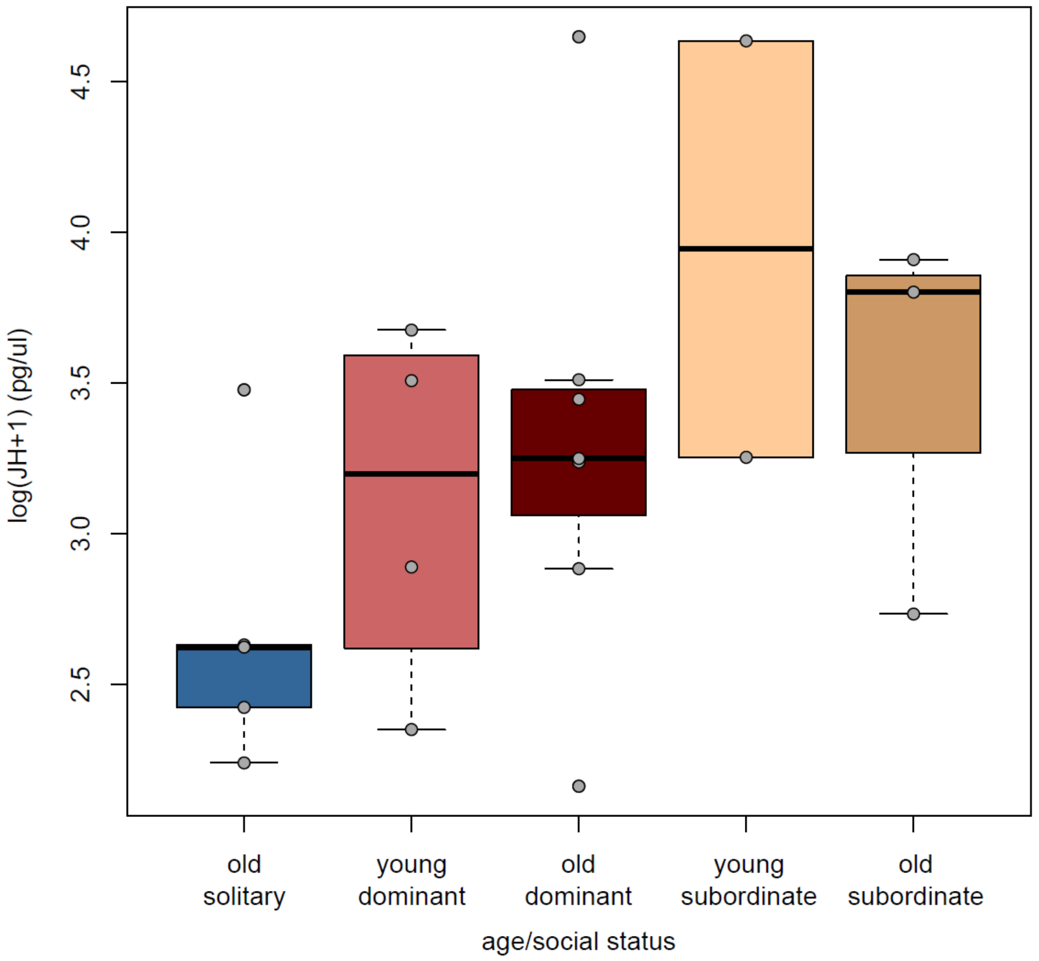

Fig. S19. Boxplot (median ± interquartile range) of log-transformed juvenile hormone titres (pg/µl) for females of different age and social status (no juvenile hormone measurements could be made for young solitary females). *E. dilemma* individuals were excluded from this analysis. Points indicate individual titres. The differences observed between groups were not statistically significant (LMM, age: $\chi^2(1,21) = 0.16$, $p = 0.69$; social status: $\chi^2(2,21) = 4.61$, $p = 0.09$).

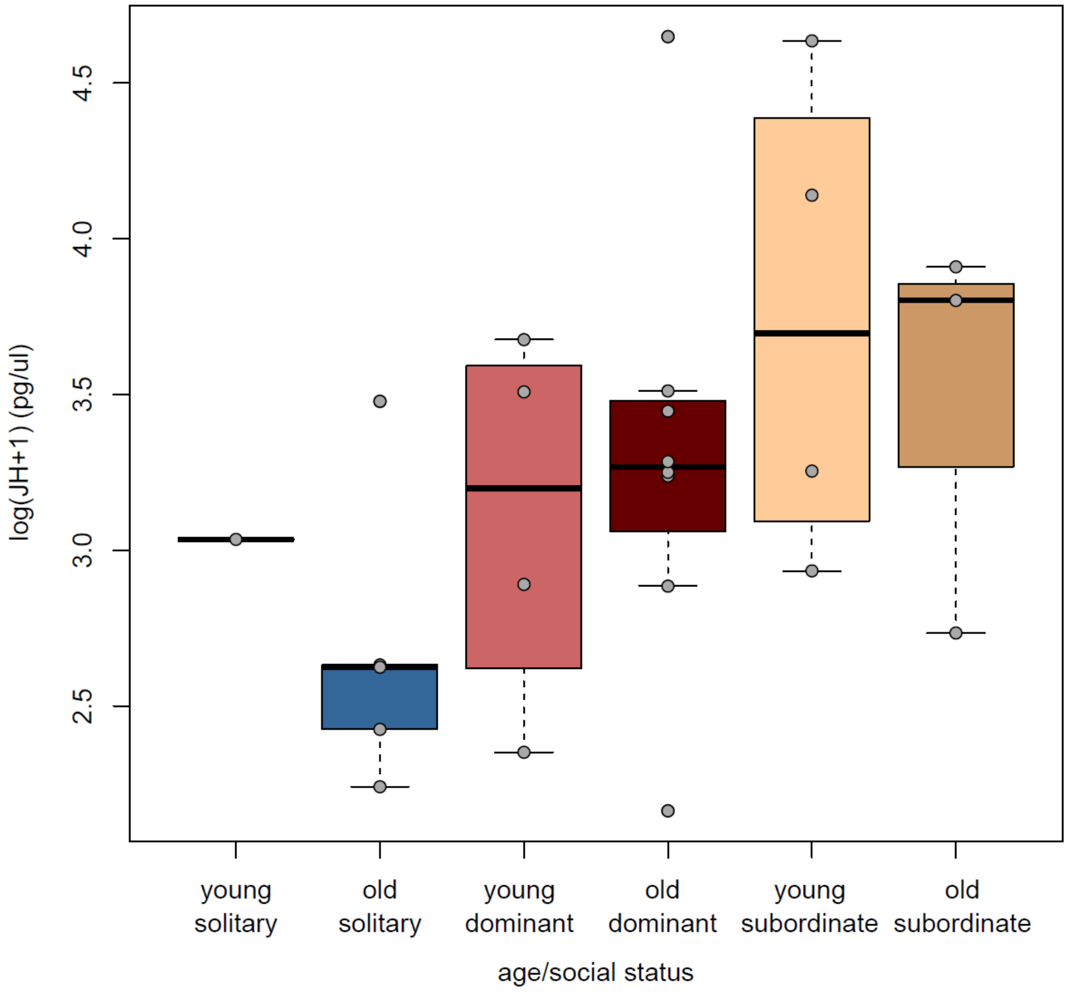

Fig. S20. Boxplot (median ± interquartile range) of log-transformed juvenile hormone titres (pg/µl) for females of different age and social status, including *E. dilemma* individuals. Points indicate individual titres. Juvenile hormone titres differed slightly according to social status when tested across all individuals, with solitary females exhibiting lower titres than females from social nests (LMM, age: $\chi^2(1,25) = 0.04$, $p = 0.84$; social status: $\chi^2(2,25) = 6.41$, $p = 0.04$).
